# Systematic literature review and meta-analysis on use of Thrombopoietic agents for chemotherapy-induced thrombocytopenia

**DOI:** 10.1371/journal.pone.0257673

**Published:** 2022-06-09

**Authors:** Gerald A. Soff, Isabelle Ray-Coquard, Luis J. Marfil Rivera, Jon Fryzek, Megan Mullins, Lauren C. Bylsma, Joseph K. Park

**Affiliations:** 1 Hematology Service, Memorial Sloan Kettering Cancer Center, New York, New York, United States of America; 2 Centre Léon Bérard, Université Claude Bernard Lyon 1, Lyon, France; 3 Servicio de Hematología, Universidad Autónoma de Nuevo León, San Nicolás de los Garza, Nuevo León, Mexico; 4 EpidStrategies, Johns Hopkins University, Rockville, Maryland, United States of America; 5 School of Public Health, University of Michigan, Ann Arbor, Michigan, United States of America; 6 EpidStrategies, Ann Arbor, Michigan, United States of America; 7 Global Development, Amgen Inc., Thousand Oaks, California, United States of America; Institute of Experimental Hematology and Transfusion Medicine, University Clinic of Bonn, GERMANY

## Abstract

**Background:**

Currently, there are no approved options to prevent or treat chemotherapy-induced thrombocytopenia (CIT). We performed a systematic literature review and meta-analysis on use of thrombopoietic agents for CIT.

**Patients and methods:**

We searched Cochrane Central Register of Controlled Trials, Cochrane Database of Systematic Reviews, PubMed, EMBASE, ClinicalTrials.gov, and health technology assessments from January 1995 to March 2021 for studies evaluating thrombopoietic agents for CIT, including recombinant human thrombopoietin (rhTPO), megakaryocyte growth and development factor (MGDF), romiplostim, and eltrombopag. Random effects meta-analyses were conducted for efficacy and safety endpoints.

**Results:**

We screened 1503 titles/abstracts, assessed 138 articles, and abstracted data from 39 publications (14 recombinant human thrombopoietin, 7 megakaryocyte growth and development factor, 9 romiplostim, 8 eltrombopag, and 1 romiplostim/eltrombopag). Random effects meta-analyses of data from multiple studies comparing thrombopoietic agents versus control (comparator, placebo, or no treatment) showed that thrombopoietic agents did not significantly improve chemotherapy dose delays and/or reductions (21.1% vs 40.4%, *P* = 0.364), grade 3/4 thrombocytopenia (39.3% vs 34.8%; *P* = 0.789), platelet transfusions (16.7% vs 31.7%, *P* = 0.111), grade ≥ 2 bleeding (6.7% vs 16.5%; *P* = 0.250), or thrombosis (7.6% vs 12.5%; *P* = 0.131). However, among individual studies comparing thrombopoietic agents with placebo or no treatment, thrombopoietic agents positively improved outcomes in some studies, including significantly increasing mean peak platelet counts (186 x 10^9^/L with rhTPO vs 122 x 10^9^/L with no treatment; *P* < 0.05) in one study and significantly increasing platelet count at nadir (56 x 10^9^/L with rhTPO vs 28 x 10^9^/L with not treatment; *P* < 0.05) in another study. Safety findings included thrombosis (*n* = 23 studies) and bleeding (*n* = 11), with no evidence of increased thrombosis risk with thrombopoietic agents.

**Conclusion:**

Our analyses generate the hypothesis that thrombopoietic agents may benefit patients with CIT. Further studies with well-characterized bleeding and platelet thresholds are warranted to explore the possible benefits of thrombopoietic agents for CIT.

## 1 Introduction

Chemotherapy-induced thrombocytopenia (CIT) is typically defined as a peripheral platelet count < 100 x 10^9^/L in patients receiving myelosuppressive chemotherapy [1,2]. CIT is common, with prevalence ranging from 21.9% to 64.2% in a retrospective cohort study of over 47,000 adult patients with cancer [3]. Overall thrombocytopenia frequency of 21.8% was reported in a separate single-institution retrospective cohort study of 614 adult patients with cancer [4].

Platelet transfusion in response to CIT is usually reserved for patients with severe thrombocytopenia (platelet count < 10 x 10^9^/L) [5]. Platelet transfusions only provide a short duration of benefit, and carry the risk of transfusion-associated adverse events including transfusion reactions, infections, and alloimmunization, which can lead to platelet transfusion refractoriness [6,7] More typically, the clinical response to CIT is to reduce the relative dose intensity (RDI) of chemotherapy, by delay and/or reduction of the chemotherapy dose. Reduced RDI may reduce treatment efficacy [8–12]. CIT may also lead to a change to less effective chemotherapy or a complete interruption of chemotherapy [9–12]. These measures may reduce the therapeutic benefits of treatments for patients with cancer, compromising patient care.

Recombinant interleukin (IL)-11 (Neumega^®^, oprelvekin) was approved for use in patients at high-risk of CIT [13]; however, its clinical use was limited due to associated side effects, including fluid retention, arrhythmias, and pulmonary edema and limited efficacy [13–16]. Because of a lack of availability of safe and effective therapy, CIT treatment remains an unmet clinical need. Targeting the thrombopoietin (TPO)/thrombopoietin receptor (TPO-R) pathway to stimulate enhanced platelet production may provide a safe and effective intervention for CIT treatment.

TPO is the primary cytokine that regulates platelet production and levels of circulating platelets [17,18]. TPO signals through the TPO-R, also known as myeloproliferative leukemia protein (MPL). TPO-R is a type I transmembrane protein that is a member of the hematopoietin/cytokine receptor superfamily [19]. Binding of TPO to the TPO-R activates the JAK/STAT and MAP kinase pathways, stimulating proliferation and maturation of committed hematopoietic progenitor cells and leading to the subsequent production of megakaryocytes and platelets [17–19].

The first generation thrombopoietic agents include recombinant human TPO (rhTPO) and a pegylated variant referred to as recombinant human megakaryocyte growth and development factor (PEG-rHuMGDF or MGDF) [20]. Second generation thrombopoietic agents bind to and activate TPO-R, but do not contain the peptide sequence of endogenous TPO. These second generation thrombopoietic agents include the peptibody romiplostim [21–23], and the small molecule agents eltrombopag [24–27], avatrombopag [28], and lusutrombopag [29] and are also referred to as thrombopoietin receptor agonists.

rhTPO has an amino acid sequence identical to that of endogenous TPO and is produced in mammalian cells [20,30]. MGDF, produced in *Escherichia coli*, includes the receptor binding 163 amino-terminal amino acids of endogenous TPO conjugated to a polyethylene glycol moiety to increase its circulation half-life [20,30]. Both rhTPO and MGDF were effective in raising platelet counts in different clinical settings [31–36]; however, their clinical development was halted following development of neutralizing antibodies against MGDF that led to persistent thrombocytopenia in some individuals [37].

Romiplostim is a fusion protein agonist of the TPO-R [21–23] approved for the treatment of thrombocytopenia in adult patients with chronic immune thrombocytopenia (ITP) who have had an insufficient response to corticosteroids, immunoglobulins, or splenectomy [38]. It is also approved for use in pediatric patients ≥ 1 year of age with ITP for ≥ 6 months who have had an insufficient response to corticosteroids, immunoglobulins, or splenectomy [38]. Romiplostim has no sequence homology to endogenous TPO [21–23]. Therefore, unlike rhTPO and MGDF, romiplostim does not illicit development of neutralizing antibodies against endogenous TPO. Romiplostim binds the distal cytokine homology region of the TPO-R, leading to increased platelet production [21,22].

Eltrombopag [24–27], avatrombopag [28], and lusutrombopag [29] are small-molecule thrombopoietic agents. Eltrombopag is approved for the treatment of thrombocytopenia in adult and pediatric patients with chronic ITP who have had an insufficient response to corticosteroids, immunoglobulins, or splenectomy and for the treatment of thrombocytopenia in patients with chronic hepatitis C, to allow the initiation and maintenance of interferon-based therapy [39]. Avatrombopag is approved for the treatment of thrombocytopenic disorders in adult patients with chronic liver disease who are scheduled to undergo a procedure and adults patients with chronic ITP who have had an insufficient response to a previous treatment [40]. Lusutrombopag is approved for thrombocytopenia in adults who are scheduled to undergo a procedure [29].

In this article, we report findings from a systematic literature review and meta-analysis on the use of first generation and second generation thrombopoietic agents for treatment or prevention of CIT. We assessed data from prospective studies designed to evaluate the efficacy and safety of the use of thrombopoietic agents in patients with CIT as well as data from retrospective studies and case series that evaluated the effectiveness and safety of thrombopoietic agents in CIT. We further determined how thrombopoietic agents compared with placebo or the standard-of-care treatments of chemotherapy dose delays and/or reductions and platelet transfusions.

## 2 Methods

### 2.1 Study search

This study was performed in accordance with PRISMA3 guidelines [41], following a pre-specified protocol. The search period was from January 1995 to March 2021. We searched the Cochrane Central Register of Controlled Trials, Cochrane Database of Systematic Reviews, PubMed, Embase, ClinicalTrials.gov, and health technology assessments (HTAs) for English-language reports of studies of thrombopoietic agents (“romiplostim OR AMG 531 OR Nplate OR Romiplate OR eltrombopag OR Promacta OR Revolade OR thrombopoietin OR TPO OR thrombopoietin receptor agonists OR thrombopoietin mimetics OR thrombopoietin stimulating agent OR megakaryocyte growth and development factor OR MGDF OR avatrombopag OR lusutrombopag”) for CIT (“chemotherapy-induced thrombocytopenia OR cancer therapy-related thrombocytopenia OR platelet transfusion”). An example of the search strategy for PubMed is shown in S1 Table. We also performed a hand search of bibliographies of articles identified as containing relevant information. Additional studies were identified from clinical input.

### 2.2 Study selection

Predefined criteria were used for study selection from the search results. We included studies that evaluated safety, efficacy (from interventional study designs), and/or effectiveness (from observational study designs) of therapies used to treat or prevent CIT. We included clinical studies, observational research, and retrospective case series. Case series of 20 or more patients were included; case series with less than 20 patients were excluded. Studies including patients receiving palliative and/or curative therapy were eligible. Duplicates were removed from the search results and the remaining publications were then independently reviewed by two reviewers in a two-part process. In part 1, the two reviewers screened titles to identify studies on the use of ≥ 1 thrombopoietic agents (rhTPO, MGDF, romiplostim, eltrombopag, avatrombopag, or lusutrombopag) in patients who had thrombocytopenia due to chemotherapy. In part 2, reviewers collected full-texts of the remaining articles and then reviewed and categorized the articles based on the presence of efficacy and safety endpoints shown in S2 Table, including chemotherapy dose delays and/or reductions, grade 3/4 thrombocytopenia, platelet transfusions, grade ≥ 2 bleeding, and thrombosis. A study was not advanced to the next stage unless both reviewers deemed it relevant. Disagreements were resolved by consensus adjudication.

### 2.3 Data extraction and synthesis

We used a systematic approach for abstracting data from the selected publications; the specific items extracted from each eligible publication are shown in S3 Table. We then summarized the characteristics and findings of the studies included in the analysis, overall and by the subgroups of solid tumors versus hematopoietic malignancies.

Endpoints of interest included time to first platelet recovery, incidence of chemotherapy dose delay by ≥ 4 days, incidence of chemotherapy dose reduction of ≥ 15% due to platelet counts < 100 x 10^9^/L, incidence of platelet transfusions, and incidence of grade ≥ 2 bleeding. These endpoints were selected as they are the endpoints being evaluated in the two ongoing phase 3 trials of romiplostim in CIT (NCT03937154 and NCT03362177).

### 2.4 Risk of bias assessment

Risk of bias in individual studies was evaluated for all included studies. Clinical trials were assessed using the Cochrane Collaboration’s ‘Risk of Bias’ tool [42] and observational studies were assessed using the Newcastle Ottawa Scale [43]. Clinical trials were evaluated for selection, performance, detection, attrition, and reporting bias and scored as “low risk,” “some concerns,” or “high risk” of bias for each domain. Observational studies were evaluated for risk of bias using the domains of selection, comparability, and outcomes on a scale ranging from 0 to 9, with 0 indicating highest risk of bias and 9 indicating lowest risk.

### 2.5 Statistical analyses

We conducted meta-analyses of data for outcomes reported in ≥ 3 studies to evaluate thrombopoietic agent versus control (comparator, placebo, or no treatment) for the prevention or treatment of CIT. We created random effects models in Comprehensive Meta-Analysis Software (Biostat, v.3.0), and then calculated summary proportions and 95% confidence intervals (CIs) of patients experiencing the outcome. We assessed heterogeneity using Cochran’s Q test and the I^2^ statistic. We created individual study weights using the inverse of their variance according to the methodology proposed by DerSimonian and Laird [44]. For the outcome of thrombosis, the types of thrombosis varied between studies; hence, a subgroup analysis was performed by thrombosis type for those outcomes reported in ≥ 3 studies. The subgroup types were venous thromboembolism (VTE) consisting of deep venous thrombosis (DVT) and/or pulmonary embolism (PE), superficial vein thrombosis (thrombophlebitis), and other (including upper extremity vein thrombosis, central venous catheter thrombosis, and myocardial infarction). We performed sensitivity analyses with studies that had thrombopoietic agent and comparator pairs only for the outcomes of chemotherapy dose delays and/or reductions, grade 3/4 thrombocytopenia, platelet transfusions, grade ≥ 2 bleeding, and thrombosis.

Publication bias was assessed for all endpoints of interest visually using funnel plots and statistically using Egger’s regression test. Where significant publication bias was present, Duval and Tweedie’s trim and fill method [45] was used as a theoretical exercise to estimate the potential impact of including unpublished estimates.

## 3 Results

### 3.1 Search results

**Fig 1:**
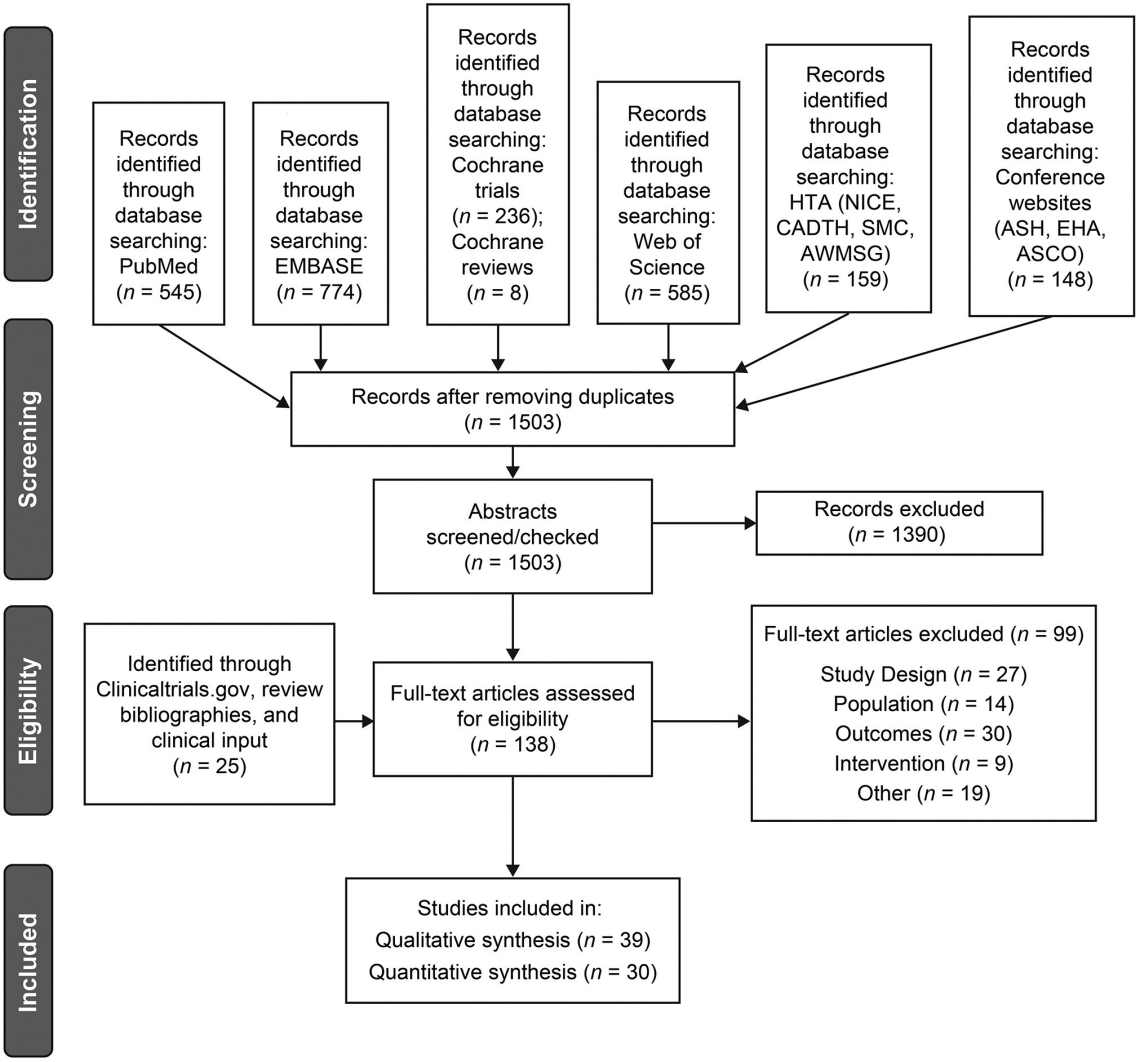
PRISMA diagram. English-language reports of studies of thrombopoietic agents identified from Cochrane Central Register of Controlled Trials, Cochrane Database of Systematic Reviews, PubMed, Embase, ClinicalTrials.gov, HTAs, a hand search of bibliographies, and clinical input were assessed for eligibility in a two-part process per a prespecified protocol to identify relevant articles for analysis. ASCO, American Society of Clinical Oncology; ASH, American Society of Hematology; AWMSG, All Wales Medicines Strategy Group; CADTH, Canadian Agency for Drugs and Technologies in Health; HTA, health technology assessment; NICE, National Institute for Health and Care Excellence; SMC, Scottish Medicines Consortium; thrombopoietic agent, thrombopoietin receptor agonist.

Results of the literature search are summarized in the PRISMA diagram (Fig 1). We identified 1503 unique English-language publications reporting studies of thrombopoietic agents after removing duplicate records from the initial searches that met the criteria for screening in the two-part process.

In part 1, two reviewers screened the titles/abstracts of the 1503 records and eliminated 1390 records due to ineligibility, leaving 113 articles for further processing. One additional relevant article Kellum et al 2010 [46] was identified from a search of the bibliography of Kuter et al 2015 [47] that had been identified from PubMed. An additional 24 records were identified through ClinicalTrials.gov and clinical input, for a combined total of 138 publications that were reviewed in part 2.

In part 2, we obtained full-text articles of the 138 records identified in part 1 and two reviewers assessed them for eligibility based on the presence of selected endpoints of interest (S3 Table). A total of 99 publications were eliminated at this stage, with reasons for elimination shown in Fig 1. The remaining 39 publications reporting results from unique studies (21 articles and 18 abstracts/posters) published from January 1995 to March 2021 were deemed eligible for assessment in this analysis.

### 3.2 Risk of bias assessment of individual studies

Among the 39 included studies, 34 were clinical trials and were evaluated using the Cochrane Risk of Bias tool [42] and 5 were observational studies and were graded using the Newcastle Ottawa Scale [43]. The clinical trials varied widely in terms of risk of bias; multiple studies received a “some concerns” designation across all 7 domains (S1 Fig). Only 3 trials received a “low risk” of bias designation across all 7 domains. Many of the trials were non-randomized or open-label and thus received “high risk” or “some concerns” for the selection bias, performance bias, and detection bias domains. The 5 observational studies had a relatively low risk of bias, with scores ranging from 6–8 on the Newcastle Ottawa Scale (S2 Fig). All observational studies were retrospective in nature and 3 did not include a non-exposed cohort.

### 3.3 Study characteristics and designs, thrombopoietic agent doses, and baseline demographics of studies that met the eligibility criteria for assessment

Study characteristics and designs, thrombopoietic agent doses administered, and baseline demographics of the patient populations in the 39 studies (a total of 2404 patients) that met the eligibility criteria for assessment are summarized in Table 1 (grouped by solid tumors and hematopoietic malignancies), with details discussed in S1 Results and presented in S4 and S5 Tables. Of the 39 studies, 30 (a total of 1973 patients) had compared a thrombopoietic agent with a control (comparator, placebo, or no treatment). Study populations most frequently had hematopoietic malignancies (*n* = 12 studies; 31.6% of studies) or non-small cell lung cancer (NSCLC) (n = 5; 13.2%), and many studies (*n* = 16; 42.1%) reported a mixture of cancers in their patient populations. The most commonly reported chemotherapy were platinum-based treatments (*n* = 18; 47.4%) and cytarabine (n = 11; 28.9%).

**Table 1:** Characteristics of studies that met the eligibility criteria for assessment.

	Thrombopoietic Agent	Study Design	Tumor Type	Comparison	Comparison Patient Number Per Study Arm
Solid tumors	rhTPO or MGDF [31–33,35,36,48–55]	14 studies: 6 RCTs and 8 non-randomized trials (*N* = 861)	NSCLC, 4 studies Gynecologic, 2 studies Sarcoma, 2 studies Breast, 1 study Mixed, 5 studies	rhIL-11	*N1* = 102 in 3 studies
				No treatment	*N1* = 352 in 5 studies
				Placebo	*N1* = 23 in 2 studies
				Other doses	*N1* = 80 in 2 studies
				None	*N1* = N/A in 2 studies
	Romiplostim [12,56–61]	7 studies: 2 RCTs, 1 non-randomized trial, and 4 case series (*N* = 322)	NSCLC, 1 study Mixed, 5 studies Breast, 1 study	None	*N1* = N/A in 4 studies
				No treatment	*N1* = 8 in 1 study
				Placebo	*N1* = 32 in 2 studies
	
	Eltrombopag [24–27,46]	5 studies: 3 RCTs and 2 non-randomized trials (*N* = 245)	Soft tissue sarcoma, 1 study Mixed, 4 studies	Placebo	*N1* = 76 in 3 studies
				None	*N1* = N/A in 1 study
				No treatment	*N1* = 3 in 1 study
	Romiplostim and eltrombopag [1]	1 retrospective case series (*N* = 27)	Glioma, 1 study	None	*N1* = N/A
Hematopoietic malignancies	rhTPO or MGDF [31,34,62–67]	8 studies: 7 RCTs and 1 non-randomized trial (*N* = 415)	AML, 4 studies NHL, 3 studies Mixed, 1 study	Placebo	*N1* = 87 in 4 studies
				Other doses	*N1* = 82 in 2 studies
				No treatment	*N1* = 117 in 2 studies
	Romiplostim [12,68–70]	3 studies: 1 RCT, 1 non-randomized trial, and 1 case series (*N* = 83)	NHL, 1 study Lymphoma, 1 study Mixed, 1 study	Placebo	*N1* = 12 in 1 study
				Other doses	*N1* = 39 in 1 study *N1* = N/A in 1 study
	Eltrombopag [12,27,71–73]	4 studies: 3 non-randomized trials and 1 RCT (*N* = 129)	AML, 3 studies Mixed, 1 study	None	*N1* = N/A in 2 studies
				Other doses	*N1* = 88 in 2 studies

*N*, total number of patients in combined studies. *N1*, total number of patients in combined subgroups of studies. AML, acute myeloid leukemia; MGDF, megakaryocyte growth and development factor; N/A, not applicable; NHL, non-Hodgkin’s lymphoma; NSCLC, non-small cell lung cancer; RCT, randomized controlled trial; rhIL-11, recombinant human interleukin 11; rhTPO, recombinant human thrombopoietin; TPO-RA, thrombopoietin receptor agonist.

### 3.4 Efficacy and safety outcomes

Results for the most frequently reported efficacy and safety outcomes for the assessed studies are discussed in S2 Results and presented in S6–S8 Tables.

Among studies comparing thrombopoietic agents with placebo, no treatment, or comparator, thrombopoietic agents were found to positively improve outcomes in some individual studies (S6 Table). In particular, thrombopoietic agents increased mean peak platelet counts and/or platelet count at nadir as summarized in S7 Table. Thrombopoietic agents significantly increased mean peak platelet counts in 3 studies: 186 x 10^9^/L with rhTPO vs 122 x 10^9^/L with no treatment (*P* < 0.05) [31], 263.9 x 10^9^/L with rhTPO vs 148.9 x 10^9^/L with no treatment (*P* < 0.05) [48], and 250.2 x 10^9^/L with rhTPO vs 160.5 x 10^9^/L with rhIL-11a (*P* < 0.05) [33] (S7 Table). Thrombopoietic agents significantly increased platelet count at nadir in a number of studies: 48 x 10^9^/L with rhTPO vs 28 x 10^9^/L with not treatment (*P* < 0.05) [35], 13 x 10^9^/L with rhTPO vs 12 x 10^9^/L with no treatment (*P* < 0.05) [31], 64.4 x 10^9^/L with rhTPO vs 52.2 x 10^9^/L with no treatment (*P* < 0.05) [48], 46.2 x 10^9^/L with rhTPO vs 37.2 x 10^9^/L with rhIL-11a (*P* < 0.05) [33], and 56 x 10^9^/L with rhTPO vs 28 x 10^9^/L with no treatment (*P* < 0.05) [49] (S7 Table). A thrombopoietic agent also significantly increased the proportion of patients who experienced platelet count correction to 100,000 μL within 3 weeks (14 of 15 patients [93.3%] vs 1 of 8 patients [12.5%]; *P* < 0.001) [56] (S7 Table), significantly decreased the duration of grade 3/4 thrombocytopenia (8 days with rhTPO vs 12 days with no treatment; *P* < 0.05) [49] and significantly decreased the proportion of patients needing transfusions (8% with MGDF vs 23% with placebo; *P* < 0.05) [62] (S6 Table). Bleeding events were reported in many of the studies (11 of 39 studies; 28.2%) (S8 Table). Thrombotic events were reported in 23 of 39 studies (59.0%), with DVT and thrombophlebitis being the most common thrombotic events (S8 Table). Overall, thrombopoietic agents appear to improve platelet outcomes without a corresponding increase in safety concerns in the individual studies.

### 3.5 Meta-analyses of efficacy and safety outcomes

**Fig 2:**
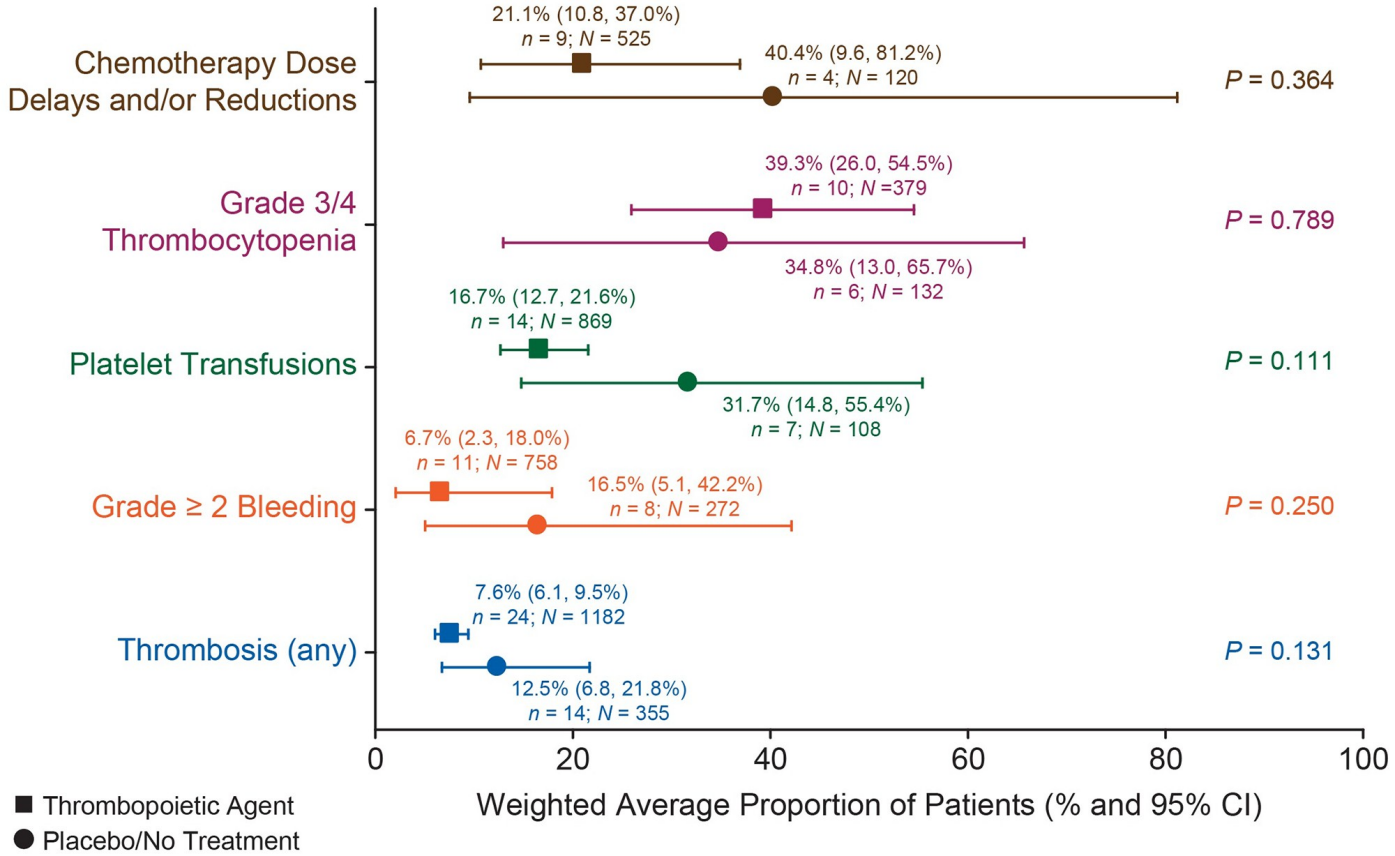
Meta-analyses of efficacy and safety outcomes. *n* = number of studies with a study arm reporting the endpoint of interest. *N* = total number of patients in study arms reporting the endpoint of interest. Meta-analyses were performed for efficacy and safety data from 30 studies that had outcomes data reported in ≥ 3 studies for thrombopoietic agents versus control (comparator, placebo, or no treatment) for the prevention or treatment of CIT across all tumor types and summary proportions (point estimates) and 95% CIs (horizontal bars) of patients experiencing the outcome calculated. Heterogeneity was assessed using Cochran’s Q test and the I2 statistic, and individual study weights created using the inverse of their variance. *n* represents the number of studies with a study arm reporting the endpoint of interest. CI, confidence interval; CIT, chemotherapy-induced thrombocytopenia; *I*^2^, degree of heterogeneity; thrombopoietic agent, thrombopoietin receptor agonist.

To quantitatively compare outcomes between thrombopoietic agent and control, we conducted meta-analyses of multiple outcomes among the thrombopoietic agent arm of each study compared with the control (comparator, placebo, or no treatment) arm, with 30 studies (a total of 1973 patients) included in the analysis. We assessed endpoints of studies if the data for the endpoint had been reported in ≥ 3 studies. Meta-analysis results for efficacy and safety outcomes of chemotherapy dose delays and/or reductions, grade 3/4 thrombocytopenia, platelet transfusions, grade ≥ 2 bleeding, and thrombosis are presented in Fig 2 and Table 2 for the multiple outcomes, and in S3 to S7 Figs by study for each outcome. We also performed sensitivity analyses with studies that had thrombopoietic agent and comparator pairs only for each outcome and the results are presented in S9 Table.

**Table 2:** Meta-analysis results for efficacy and safety outcomes for thrombopoietic agent versus placebo or no treatment by combined and individual thrombopoietic agents.

Analysis	Rate in Intervention Group^a^			Rate in Comparison Group^a^			*P*-value
	*n*	Rate (95% CI)	*P*-Het; *I*^2^	*n*	Rate (95% CI)	*P*-Het; *I*^2^	
Chemotherapy dose delays and/or reductions	9	21.1% (10.8%, 37.0%)	< 0.001; 89.3%	4	40.4% (9.6%, 81.2%)	< 0.001; 91.0%	0.364
Grade 3/4 thrombocytopenia	10	39.3% (26.0%, 54.5%)	< 0.001; 84.4%	6	34.8% (13.0%, 65.7%)	< 0.001; 82.7%	0.789
Platelet transfusion	14	16.7% (12.7%, 21.6%)	0.005; 56.0%	7	31.7% (14.8%, 55.4%)	0.001; 74.6%	0.111
Grade ≥ 2 bleeding	11	6.7% (2.3%, 18.0%)	< 0.001; 92.4%	8	16.5% (5.1%, 42.2%)	< 0.001; 75.3%	0.250
Thrombosis (any)^b^	24	7.6% (6.1%, 9.5%)	0.65; 0%	14	12.5% (6.8%, 21.8%)	0.12; 31.8%	0.131
Deep venous thrombosis	9	5.4% (3.5%, 8.4%)	0.30; 16.2%	3	33.3% (11.1%, 66.7%)	0.99; 0%	0.004
Pulmonary embolism	11	3.5% (2.1%, 5.8%)	0.82; 0%	5	25.3% (8.3%, 55.7%)	0.99; 0%	0.002
Thrombophlebitis	3	2.6% (0.8%, 7.8%)	0.93; 0%	3	3.6% (0.2%, 38.4%)	0.99; 0%	0.838
Other^c^	8	3.1% (1.6%, 5.8%)	0.21; 27.3%	4	5.4% (2.0%, 13.5%)	0.99; 0%	0.340

*n* = number of studies with a study arm reporting the endpoint of interest.

^a^The rate in comparison group for each thrombopoietic agent is the meta-analysis for the comparison groups in studies that evaluated each thrombopoietic agent only. For example, for eltrombopag, the rate in the intervention group is the rate among all studies with an eltrombopag arm; the rate in the comparison group is the rate among those eltrombopag studies but only in the comparison arm, it does not include comparison arms of studies evaluating other thrombopoietic agents.

^b^The overall measure of thrombosis reported in studies. Subgroups do not add up to 51 as some studies reported multiple types of thrombotic events, which were included as a summary measure in the overall thrombosis analysis.

^c^Specific types of thrombotic events reported in < 3 studies (insufficient number for a meta-analysis); includes subclavian vein thrombosis, central venous catheter thrombosis, portal vein thrombosis, renal vein thrombosis, myocardial infarction, and cerebrovascular accident.

CI, confidence interval; Het, heterogeneity; I^2^, degree of heterogeneity; MGDF, megakaryocyte growth and development factor; N/A, not applicable; rhTPO, recombinant human thrombopoietin.

#### 3.5.1 Chemotherapy dose delays and/or reductions

Data from the thrombopoietic agent arms of 9 studies [9,25,26,50,56–58,62,71] and control arms of 4 studies [25,26,62,71] were included in the meta-analysis for chemotherapy dose delays and/or reductions (Fig 2, Table 2, S3 Fig). Although there is a favorable trend, thrombopoietic agents did not significantly decrease dose delays and/or reductions compared with placebo or no treatment (21.1% [95% CI: 10.8%, 37.0%] vs 40.4% [95% CI: 9.6%, 81.2%], *P* = 0.364) (Fig 2, Table 2). Results with data meta-analyzed by study are shown in S3 Fig.

#### 3.5.2 Grade 3/4 thrombocytopenia

Data from the thrombopoietic agent arms of 10 studies [24–26,34,51,57,62,64,68,69,71] and control arms of 6 studies [24–26,57,62,71] were included in the meta-analysis for grade 3/4 thrombocytopenia (Fig 2, Table 2, S4 Fig). Rates of grade 3/4 thrombocytopenia appeared to be similar for thrombopoietic agents and placebo or no treatment (39.3% vs 34.8%; *P* = 0.789) (Fig 2, Table 2). Results with data meta-analyzed by study are shown in S4 Fig.

#### 3.5.3 Platelet transfusions

Data from the thrombopoietic agent arms of 14 studies [9,25,32,33,35,50,52,56,57,62,64,68–70,74] and control arms of 7 studies [25,32,35,56,57,62,70] were included in the meta-analysis for platelet transfusions (Fig 2, Table 2, S5 Fig). Rates of platelet transfusions were not significantly lower with thrombopoietic agents than with control (16.7% [95% CI: 12.7%, 21.6%] vs 31.7% [95% CI: 14.8%, 55.4%], *P* = 0.111) (Fig 2, Table 2). Results with data meta-analyzed by study are shown in S5 Fig.

#### 3.5.4 Grade ≥ 2 bleeding

Data from the thrombopoietic agent arms of 11 studies [9,24,25,35,46,50,57,63,64,68,69,71] and control arms of 8 studies [24,25,35,46,50,57,63,71] were included in the meta-analysis of grade ≥ 2 bleeding (Fig 2, Table 2, S6 Fig). Similar to platelet transfusions, rates of bleeding were not significantly lower with thrombopoietic agents than with control (6.7% [95% CI: 2.3%, 18.0%] vs 16.5% [95% CI: 5.1%, 42.2%], *P* = 0.250) (Fig 2, Table 2). Results with data meta-analyzed by study are shown in S6 Fig.

#### 3.5.5 Thrombosis

Data from the thrombopoietic agent arms of 24 studies [1,9,24–26,32,35,36,46,50,53,56–61,63–66,68–71] and control arms of 14 studies [24–26,32,35,36,46,50,57,63,65,66,70,71] were included in the meta-analysis for thrombosis (Fig 2, Table 2, S7 Fig). There was no increased risk of thrombosis with thrombopoietic agents (7.6% [95% CI: 6.1%, 9.5%] vs 12.5% [95% CI: 6.8%, 21.8%], *P* = 0.131) (Fig 2, Table 2). Results with data meta-analyzed by study are shown in S7 Fig. Meta-analysis results by subgroups of thrombosis type (Table 2) showed a significantly decreased risk with thrombopoietic agents than with placebo or no treatment for DVT (*P* = 0.004) and PE (*P* = 0.002) but no changes in risk for thrombophlebitis (*P* = 0.838) and unspecified thrombosis (*P* = 0.340).

#### 3.5.6 Sensitivity analyses including only studies with thrombopoietic agent/comparator pairs

The sensitivity analyses with studies that had thrombopoietic agent and comparator pairs only showed similar results to the original analysis, with no statistically significant differences between the thrombopoietic agent and comparator for efficacy and safety outcomes of chemotherapy dose delays and/or reductions, grade 3/4 thrombocytopenia, platelet transfusions, grade ≥ 2 bleeding, and thrombosis (any) (S9 Table). Also similar to the original assays, the sensitivity analyses showed a significantly decreased risk with thrombopoietic agents than with comparator for DVT (*P* = 0.042) and PE (*P* = 0.006) but no changes in risk for thrombophlebitis (*P* = 0.838) and unspecified thrombosis (*P* = 0.340) (S9 Table).

#### 3.5.7 Publication bias

For all endpoints of interest, the potential for publication bias was assessed using funnel plots and Egger’s regression test. Egger’s regression test and visual inspection of the funnel plots did not demonstrate significant publication bias for the outcomes of chemotherapy dose delays and/or reductions, grade 3/4 thrombocytopenia, platelet transfusions, or thrombosis (S8 Fig, panels A–C, E). However, funnel plot asymmetry and significant Egger’s regression test (*P* = 0.024) was present for the endpoint of grade ≥ 2 bleeding (S8 Fig, panel D), indicating that publication bias was likely present. The funnel plot indicated that the effect resulted in an underestimation of bleeding events for thrombopoietic agents. Imputation of theoretically missing studies to the right of the mean using Duval and Tweedie’s trim and fill method resulted in an adjusted estimate of 10.3% (95% CI: 4.18–23.18%) for bleeding events in the thrombopoietic agent arms, slightly higher than the 6.7% [95% CI: 2.3%, 18.0%] seen in the original analysis for the thrombopoietic agent arms.

## 4 Discussion

In our literature search and analysis, we identified 39 studies that had evaluated the use of thrombopoietic agents in the prevention or treatment of CIT. Thrombopoietic agents in CIT have been evaluated in both solid tumors (mostly NSCLC, 5 studies) and hematopoietic malignancies (12 studies) and with different chemotherapies, mostly platinum based (18 studies) or chemotherapies containing cytarabine (11 studies).

Our qualitative analysis of data from identified individual studies showed that in some studies thrombopoietic agents compared with a control (comparator, placebo, or no treatment) significantly increased mean peak platelet counts [31,33,48] and significantly increased platelet counts at nadir [31,33,35,48,49]. Thrombopoietic agents also significantly decreased the duration of grade 3/4 thrombocytopenia [49] and significantly decreased the proportion of patients needing transfusions [62]. A recent randomized phase 2 controlled trial [56] showed the benefit of romiplostim in correcting CIT, with more patients in the romiplostim vs the observation group experiencing platelet count correction to 100,000 μL (14 of 15 patients [93.3%] vs 1 of 8 patients [12.5%], *P* < 0.001) within 3 weeks. In an extension of that study, patients who achieved platelet correction with romiplostim resumed chemotherapy with only 6.8% experiencing recurrent reduction or delay of chemotherapy due to CIT [56].

We quantitatively assessed the endpoints of studies comparing thrombopoietic agent arms and control arms (comparator, placebo, or no treatment), if the data for the endpoint had been reported in ≥ 3 studies. Efficacy endpoints that met the criteria included chemotherapy dose delays and/or reductions, grade 3/4 thrombocytopenia, platelet transfusions, grade ≥ 2 bleeding, and thrombosis. Allowing for a range in study designs, both first- and second-generation thrombopoietic agents showed similar results. Thrombopoietic agent use for the treatment of CIT was associated with a nonsignificant reduction in chemotherapy dose delays and/or reductions (21.1% vs 40.4%, *P* = 0.364). Bleeding rates were difficult to compare due to varying definitions amongst studies; taking this into account, grade ≥ 2 bleeding showed a nonsignificant reduction in thrombopoietic agent use (6.7% vs 16.5%, *P* = 0.250). Similarly, nonsignificant improvements with use of thrombopoietic agents were observed for grade 3/4 thrombocytopenia (39.3% vs 34.8%; *P* = 0.789), platelet transfusions (16.7% vs 31.7%, *P* = 0.111), and thrombosis (7.6% vs 12.5%; *P* = 0.131). Of note, doses and dosing schedules for each thrombopoietic agent varied widely across the studies. There are currently insufficient data in the literature to allow for assessment of optimal doses or scheduling of thrombopoietic agents, and to allow for assessment of relative efficacy of the different thrombopoietic agents. Additionally, publication bias was likely present for the endpoint of grade ≥ 2 bleeding but not for the other endpoints.

The most common safety endpoint was incidence of thrombosis, reported in 23 of 39 studies. Thrombosis, in general, is a major concern in patients with cancer receiving chemotherapy. Studies have indicated that thrombopoietic agents may increase the risk of thrombosis in adult patients with ITP [75,76]. Data from our analysis, however, did not show an increased risk of thrombosis with use of thrombopoietic agents.

Our literature search did not identify studies evaluating the cost-effectiveness of thrombopoietic agents in CIT. However, a recent study [77] reported on the cost and risks of CIT in patients identified using an algorithm that included use of a thrombopoietic agent. The retrospective study used data from two US private healthcare databases to evaluate the incidence, clinical consequences, and economic costs in 215,508 adult patients who had received chemotherapy for solid tumors or non-Hodgkin’s lymphoma. The study reported a 9.7% incidence rate of CIT, with a third of CIT episodes managed in the hospital. The mean length of hospital stay was 4.6 days and the mean cost of inpatient care was $36,448 for a first-listed CIT diagnosis, with a mean cost of CIT-related care across cycles of $2179 per patient episode [77]. More studies specifically evaluating the cost of thrombopoietic agents compared with the cost of CIT-related care are warranted.

Findings from our literature search and analysis generally point to the benefit of using thrombopoietic agents for the prevention or treatment of CIT. This is in contrast to the findings from a similar analysis of data from studies in patients with solid tumors that reported no evidence to support the use of thrombopoietic agents in preventing or treating CIT [2]. That earlier analysis [2] assessed data from a limited sample population of 268 patients with solid tumors enrolled in 3 randomized controlled trials, to evaluate the effect of thrombopoietic agents compared with placebo on bleeding events, overall survival (primary outcome), and quality of life. The authors could not draw any certain conclusions from the analysis due to lack of strong evidence because of limited data [2]. In contrast, our analysis evaluated data from studies that had enrolled patients with solid tumors or hematopoietic malignancies, and also included data from randomized controlled trials as well as case series reviews and observational studies, with 39 studies (a total of 2404 patients) included in the qualitative analysis part and 30 studies (a total of 1973 patients) included in the quantitative analysis part. We also did not evaluate overall survival or quality of life. As such, the difference in the total number of studies, total number of patients, cancer types, and outcomes evaluated may have contributed to the differences in findings between our analysis and the analysis conducted earlier [2].

The advantage of our current literature review and meta-analysis is that it allowed a comparison of outcomes data generated to date between thrombopoietic agents and placebo or observation in CIT, to understand the current literature landscape. However, several limitations must be taken into consideration. First, the literature review was limited to reports (abstracts and articles) published in English and did not include reports in other languages. Secondly, varying agents, doses, and regimens (particularly for timing of dosing) were reported in the different studies. Thirdly, data for only a few endpoints of interest were reported for any of the studies evaluated; thus, data for any particular endpoint were available from only a few studies. Also, definitions for some of the outcomes differed across the studies. Survival data, which would be considered a definitive outcome measure of the benefit of thrombopoietic agents, was reported in only two studies [62,66] and quality of life (an episode of depression) was reported in only one study [57,78], limiting the analysis that could be performed for these outcomes.

In conclusion, findings from our literature review and analysis generally point to the benefit of using thrombopoietic agents for the prevention or treatment of CIT. Meta-analysis of results from multiple studies to compare outcomes between thrombopoietic agents and controls showed that thrombopoietic agents had no significant effect on chemotherapy dose delays and/or reductions, grade 3/4 thrombocytopenia, platelet transfusions, grade ≥ 2 bleeding, or thrombosis. However, among individual studies comparing thrombopoietic agents with placebo, no treatment, or comparator, thrombopoietic agents significantly improved platelet responses. The favorable outcomes in platelet responses in individual studies generate the hypothesis that thrombopoietic agents may generally improve outcomes in secondary prevention of CIT. Further study with well-characterized bleeding and platelet thresholds is needed to explore the possible benefits of thrombopoietic agents for CIT compared with current care options of platelet transfusions or chemotherapy dose delays and/or reductions. Currently, two phase 3 trials evaluating romiplostim in CIT (NCT03937154 and NCT03362177) are in progress.

## Supporting information

S1 Checklist (PDF)

S1 Fig Risk of bias assessment of clinical studies included in analysis. (PDF)

S2 Fig Risk of bias assessment of observational studies included in analysis. (PDF)

S3 Fig Meta-analysis of data for chemotherapy dose delays and/or reductions by study. (PDF)

S4 Fig Meta-analysis of data for grade 3/4 thrombocytopenia by study. (PDF)

S5 Fig Meta-analysis of data for platelet transfusions by study. (PDF)

S6 Fig Meta-analysis of data for grade ≥ 2 bleeding by study. (PDF)

S7 Fig Meta-analysis of data for thrombosis (any) by study. (PDF)

S8 Fig Funnel plot for publication bias for chemotherapy dose delays and/or reductions (A), grade 3/4 thrombocytopenia (B), platelet transfusions (C), grade ≥ 2 bleeding (D), and thrombosis (any) (E) in thrombopoietic agent arms of included studies. (PDF)

S1 Table Example of search strategy. (PDF)

S2 Table Study selection endpoints. (PDF)

S3 Table Specific items extracted from each selected publication. (PDF)

S4 Table Detailed characteristics of studies that met the eligibility criteria for assessment by thrombopoietic agent type and publication year. (PDF)

S5 Table Baseline characteristics of patient populations in the assessed studies by thrombopoietic agent type and publication year. (PDF)

S6 Table Efficacy outcomes by thrombopoietic agent type and publication year. (PDF)

S7 Table Platelet outcomes for thrombopoietic agent versus control by publication year. (PDF)

S8 Table Safety outcomes by thrombopoietic agent type and publication year. (PDF)

S9 Table Sensitivity analyses including studies with thrombopoietic agent/comparator pairs only. (PDF)

S1 Results Study characteristics and designs, thrombopoietic agent doses, and baseline demographics of studies that met the eligibility criteria for assessment. (PDF)

S2 Results Efficacy and safety outcomes. (PDF)
