# Supplementary material for: Systematic literature review and meta-analysis on use of Thrombopoietic agents for chemotherapy-induced thrombocytopenia
Source: PLoS One. 2022 Jun 9;17(6):e0257673. doi: 10.1371/journal.pone.0257673 (PMC9183450; doi:10.1371/journal.pone.0257673)
Supplement: S2 Fig — (PDF) [file pone.0257673.s003.pdf]

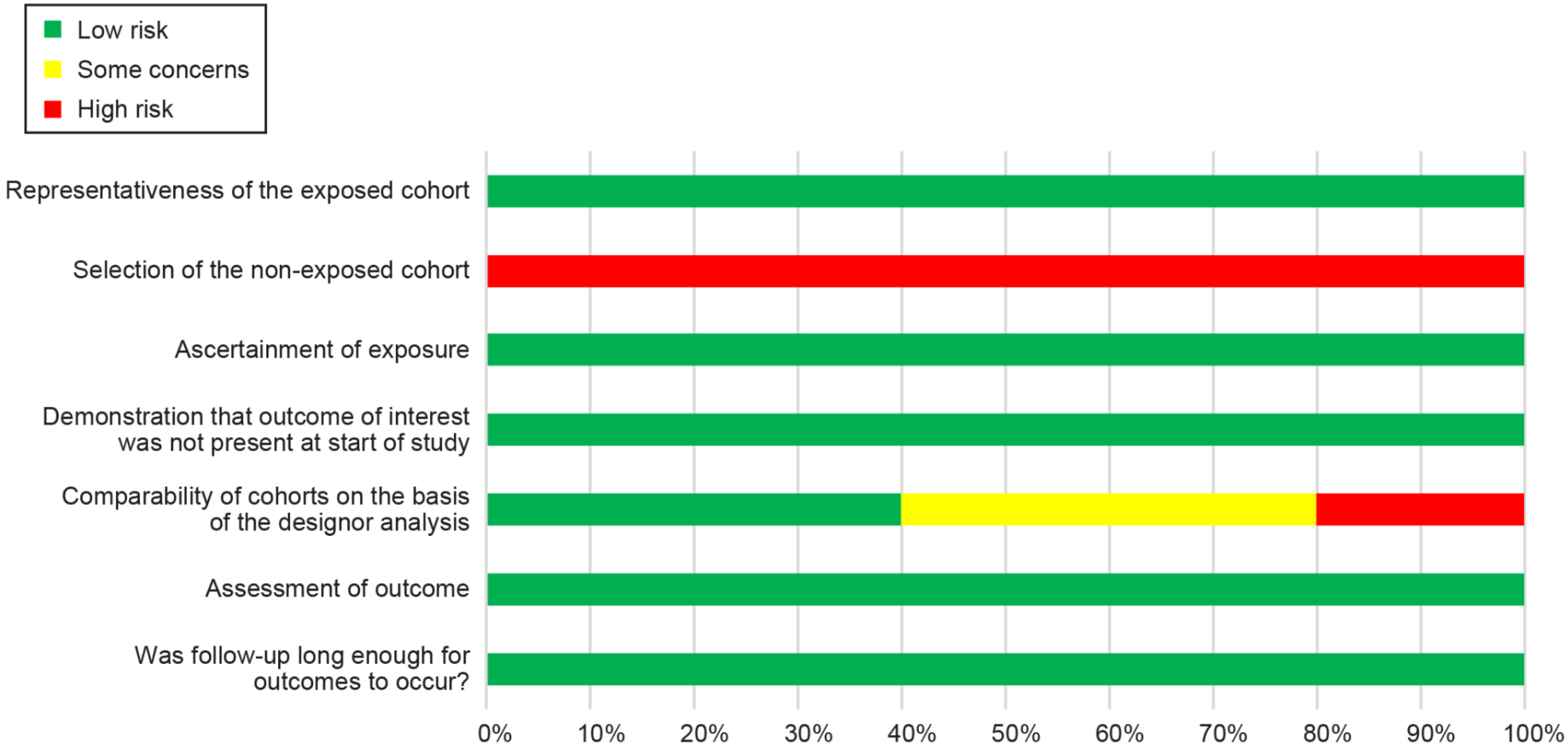

S2 Fig. Risk of bias assessment of observational studies included in analysis. Observational studies were evaluated for risk of bias using the domains of selection, comparability, and outcomes on a scale ranging from 0 to 9, with 0 indicating highest risk of bias and 9 indicating lowest risk.
