# Supplementary material for: Systematic literature review and meta-analysis on use of Thrombopoietic agents for chemotherapy-induced thrombocytopenia
Source: PLoS One. 2022 Jun 9;17(6):e0257673. doi: 10.1371/journal.pone.0257673 (PMC9183450; doi:10.1371/journal.pone.0257673)
Supplement: S4 Fig — (PDF) [file pone.0257673.s005.pdf]

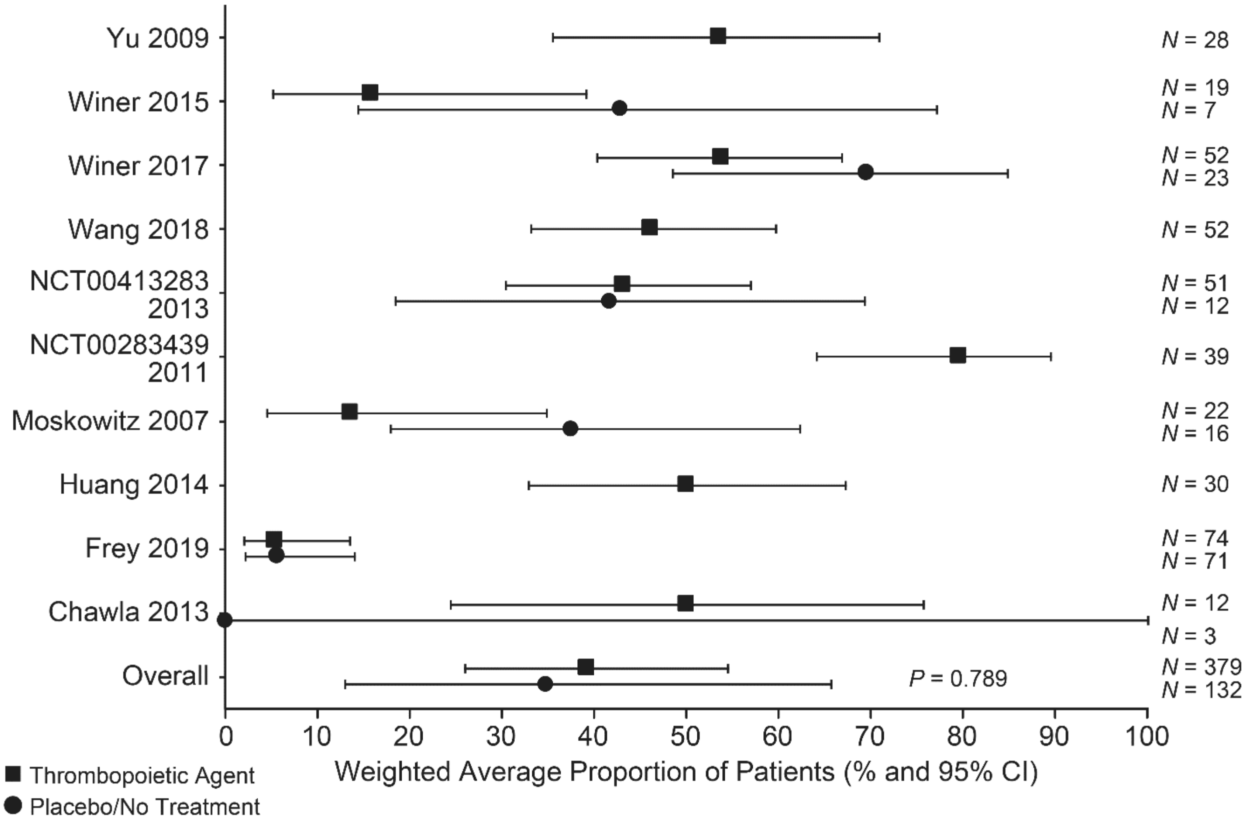

S4 Fig. Meta-analysis of data for grade 3/4 thrombocytopenia by study. *N* = number of patients in study arm. Meta-analysis of data from 10 studies that had reported grade 3/4 thrombocytopenia were performed and summary proportions (point estimates) and 95% CIs (horizontal bars) of patients experiencing the outcome calculated. CI, confidence interval.
