# Supplementary material for: Systematic literature review and meta-analysis on use of Thrombopoietic agents for chemotherapy-induced thrombocytopenia
Source: PLoS One. 2022 Jun 9;17(6):e0257673. doi: 10.1371/journal.pone.0257673 (PMC9183450; doi:10.1371/journal.pone.0257673)
Supplement: S7 Fig — (PDF) [file pone.0257673.s008.pdf]

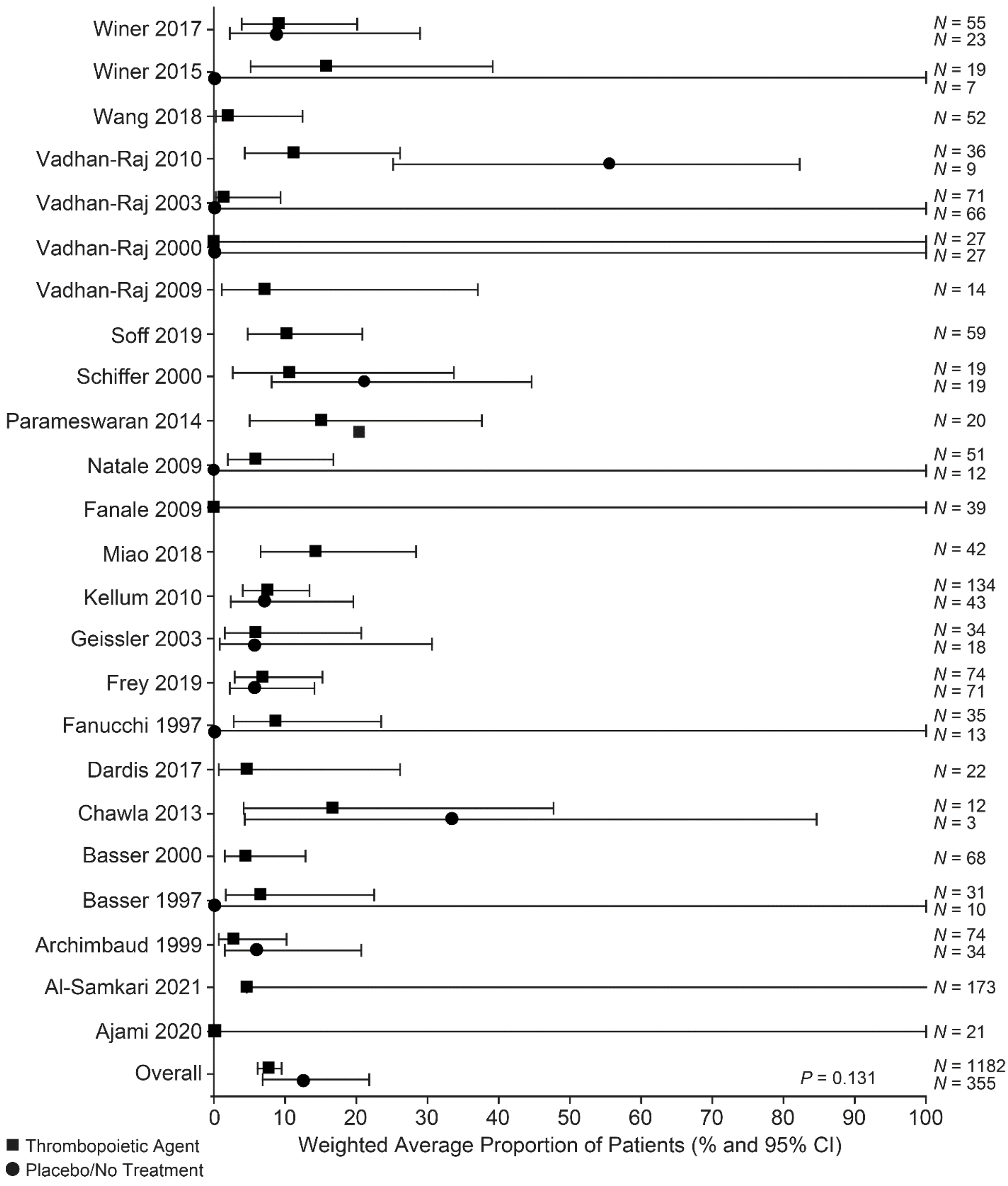

S7 Fig. Meta-analysis of data for thrombosis (any) by study. *N* = number of patients in study arm. Meta-analysis of data from 24 studies that had reported thrombosis were performed and summary proportions (point estimates) and 95% CIs (horizontal bars) of patients experiencing the outcome calculated. CI, confidence interval.
