# Supplementary material for: Systematic literature review and meta-analysis on use of Thrombopoietic agents for chemotherapy-induced thrombocytopenia
Source: PLoS One. 2022 Jun 9;17(6):e0257673. doi: 10.1371/journal.pone.0257673 (PMC9183450; doi:10.1371/journal.pone.0257673)
Supplement: S8 Fig — (PDF) [file pone.0257673.s009.pdf]

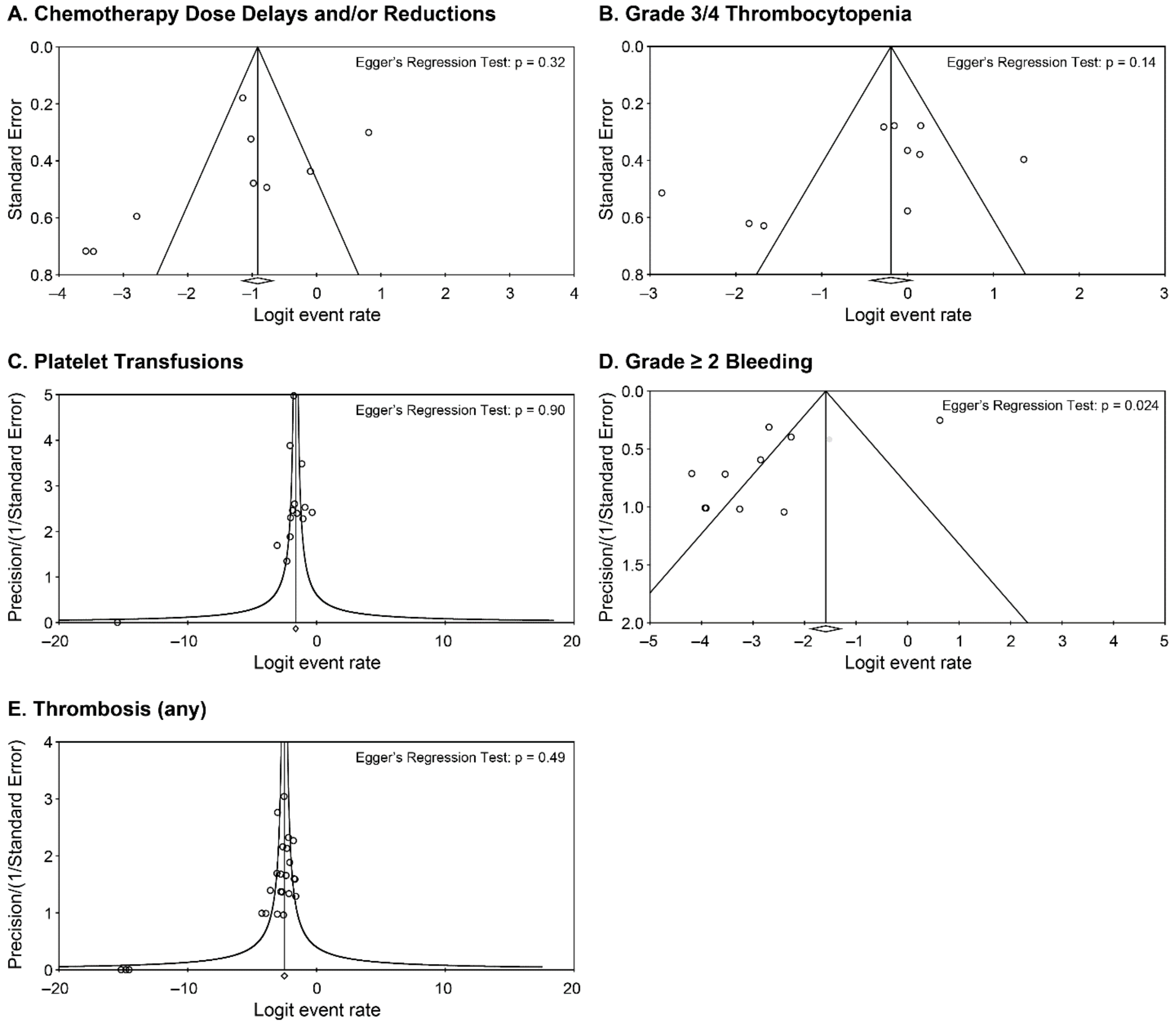

S8 Fig. Funnel plots for publication bias for chemotherapy dose delays and/or reductions (A), grade 3/4 thrombocytopenia (B), platelet transfusions (C), grade ≥ 2 bleeding (D), and thrombosis (any) (E) in thrombopoietic agent arms of included studies. The funnel plots present the proportion of patients achieving each outcome in the thrombopoietic agent arm of each study by the standard error. Visual asymmetry is present in the funnel plot with most of the studies to the left of the mean for grade ≥ 2 bleeding but not for the other outcomes.
