# Supplementary material for: Systematic literature review and meta-analysis on use of Thrombopoietic agents for chemotherapy-induced thrombocytopenia
Source: PLoS One. 2022 Jun 9;17(6):e0257673. doi: 10.1371/journal.pone.0257673 (PMC9183450; doi:10.1371/journal.pone.0257673)
Supplement: S1 Table — (PDF) [file pone.0257673.s010.pdf]

S1 Table. Example of the PubMed search strategy

Number	PubMed Search
1	romiplostim OR AMG 531 OR Nplate OR Romiplate
2	eltrombopag OR Promacta OR Revolade
3	thrombopoietin OR TPO
4	thrombopoietin receptor agonists OR thrombopoietin mimetics OR thrombopoietin stimulating agent
5	megakaryocyte growth and development factor OR MGDF
6	1 OR 2 OR 3 OR 4 OR 5
7	chemotherapy-induced thrombocytopenia OR cancer therapy-related thrombocytopenia OR chemotherapy-associated thrombocytopenia
8	platelet transfusion
9	7 OR 8
10	6 AND 9
