# Supplementary material for: Systematic literature review and meta-analysis on use of Thrombopoietic agents for chemotherapy-induced thrombocytopenia
Source: PLoS One. 2022 Jun 9;17(6):e0257673. doi: 10.1371/journal.pone.0257673 (PMC9183450; doi:10.1371/journal.pone.0257673)
Supplement: S2 Table — (PDF) [file pone.0257673.s011.pdf]

S2 Table. Study selection endpoints

Endpoint	Definition
Efficacy	▪ Platelet-related such as platelet count, platelet response (≥ 100 x 10^9/L), time to first platelet recovery (≥ 100 x 10^9/L), presence of dose-limiting CIT, grade 3/4 thrombocytopenia and duration of such thrombocytopenia, platelet nadir, days with platelet counts of < 100 x 10^9/L<br>▪ Platelet transfusions, both incidence and number of units<br>▪ Chemotherapy-related, i.e., resumption of chemotherapy, days of delay (including incidence of delay by ≥ 4 days), dose intensity/chemotherapy dose reductions (including dose reductions of ≥ 15% due to thrombocytopenia), switching chemotherapy regimens
Survival	▪ Overall survival (as available, possible measures include median survival, 1-year survival rate, 5-year survival rate, early death [< 30 days after treatment initiation], hazard ratios), and progression/relapse-free survival
Safety	▪ For example, grade ≥ 2 bleeding (if a full list of terms was provided, they were checked against the haemorrhage/hemorrhage MedDRA list as available at http://purl.bioontology.org/ontology/MEDDRA/10055798), thrombotic events, progression of cancer
Other	▪ Any PRO/QoL data and what PRO and/or QoL instruments were used<br>▪ Any cost-effectiveness data and how the data were obtained<br>▪ If the study was population-based (vs hospital-based), any data on the incidence and prevalence of CIT were noted and categorized in 5-year buckets (e.g., 1995 to 2000, 2000 to 2005, etc.), as the incidence and prevalence may change over time.

CIT, chemotherapy-induced thrombocytopenia; MedDRA, Medical Dictionary for Regulatory Activities; PRO, patient-reported outcome; QoL, quality of life.
