# Supplementary material for: Systematic literature review and meta-analysis on use of Thrombopoietic agents for chemotherapy-induced thrombocytopenia
Source: PLoS One. 2022 Jun 9;17(6):e0257673. doi: 10.1371/journal.pone.0257673 (PMC9183450; doi:10.1371/journal.pone.0257673)
Supplement: S3 Table — (PDF) [file pone.0257673.s012.pdf]

S3 Table. Specific items extracted from each selected publication

Extracted Items
▪ General information, including title, authors, source, contact address, country
▪ Year of publication
▪ Trial characteristics, including details of interventions/comparators, study design and duration, definition of thrombocytopenia, chemotherapy administered
▪ Participant demographics and disease characteristics (including reporting of key confounding factors)
▪ Efficacy outcomes (listed in S1 Table)
▪ Safety outcomes (listed in S1 Table)
▪ Quality assessment of study reviewed via Cochrane Risk of Bias Tool
