# Supplementary material for: Systematic literature review and meta-analysis on use of Thrombopoietic agents for chemotherapy-induced thrombocytopenia
Source: PLoS One. 2022 Jun 9;17(6):e0257673. doi: 10.1371/journal.pone.0257673 (PMC9183450; doi:10.1371/journal.pone.0257673)
Supplement: S4 Table — (PDF) [file pone.0257673.s013.pdf]

S4 Table. Detailed characteristics of studies that met the eligibility criteria for assessment by thrombopoietic agent type and publication year

Study Authors, Year	Location	Study Design	Intervention (*N*)	Intervention Dose	Comparison (*N*)	Tumor Type	Chemotherapy	Relevant Outcomes	Meta-analysis (Yes/No)
**First-generation thrombopoietic agents**									
Vadhan-Raj et al, 1997 [74]	USA	Phase 1/2 non-randomized trial	rhTPO (12)	0.3, 0.6, 1.2, and 2.4 μg/kg as a single dose 3 weeks before chemotherapy start	Other doses in trial (12)	Sarcoma	NR	Baseline platelet count, peak platelet count	No
Vadhan-Raj et al, 2000 [35]	USA	Phase 1/2 trial	rhTPO (27)	0.6, 1.2, 2.4, and 3.6 mg/kg/day	No treatment (27)	Gynecologic cancer	Carboplatin	Baseline platelet count, platelet response, platelet nadir, transfusions, bleeding, thrombosis	Yes
Vadhan-Raj et al, 2001 [75]	USA	Feasibility trial	rhTPO (20)	1.2 μg/kg on Days 1 and 4	None (N/A)	Ovarian cancer	Carboplatin	Peak platelet count	No
Vadhan-Raj et al, 2003 [58]	USA	Phase 1/2 crossover trial	rhTPO (66)	1.2, 2.4, or 3.6 μg/kg; varied dosing schedules	No treatment (66)	Sarcoma	Doxorubicin + ifosfamide	Peak platelet count, platelet response, platelet nadir, days $< 100 \times 10^9$/L, transfusions, dose delays/reductions, bleeding, thrombosis	Yes
Bai, Zou et al, 2004 [31]	China	Crossover trial	rhTPO (81)	1 μg/kg/day, 6–24 hours after chemotherapy	No treatment (81)	Solid tumors and leukemia	NR	Peak platelet count, platelet nadir	No

Study Authors, Year	Location	Study Design	Intervention (*N*)	Intervention Dose	Comparison (*N*)	Tumor Type	Chemotherapy	Relevant Outcomes	Meta-analysis (Yes/No)
Bai, Xu et al, 2004 [48]	China	Randomized trial	rhTPO (154)	1 µg/kg/day, 6–24 hours after chemotherapy	No treatment (154)	Solid tumors	NR	Peak platelet count, platelet nadir	No
Dai et al, 2008 [33]	China	Non-randomized trial	rhTPO (35)	15,000 U/day	rhIL-11 (37)	Solid tumors	NR	Peak platelet count, platelet nadir, transfusions	Yes
Yu et al, 2009 [61]	China	RCT	rhTPO (28)	15,000 U/day	rhIL-11 (34)	NSCLC	Gemzar + cisplatin	Peak platelet count, grade 3/4 thrombocytopenia, platelet nadir	Yes
Xu et al, 2011 [49]	China	Crossover trial	rhTPO (24)	300 U/kg/day on Days 2, 4, 6, and 9	No treatment (24)	NSCLC	NR	Platelet nadir	No
Huang et al, 2014 [34]	China	Crossover trial	TPO (30) on Days 2-11	15,000 U/day	TPO (30) on Days –4, –2, 2–9	NHL	Cytarabine	Platelet response, grade 3/4 thrombocytopenia, platelet nadir	Yes
Sui et al, 2017 [76]	NR	Prospective RCT	rhTPO (49)	15,000 U/day starting dose when platelet count was ≤ 50 x 10^9/L until platelet count increase to > 100 x 10^9/L or for 21 days	No treatment (36)	AML	Daunorubicin, mitoxantrone, cytarabine	Peak platelet count, platelet response, transfusions	No

Study Authors, Year	Location	Study Design	Intervention (*N*)	Intervention Dose	Comparison (*N*)	Tumor Type	Chemotherapy	Relevant Outcomes	Meta-analysis (Yes/No)
Wang et al, 2018 [60]	China	Crossover trial	rhTPO (52)	10 doses at 15,000 U/dose either before or after chemotherapy	Other dosing schedule of rhTPO (52)	NHL	Cytarabine + cisplatin + dexamethasone (30); cytarabine + ifosfamide + etoposide (11); cytarabine + methotrexate (11)	Grade 3/4 thrombocytopenia, platelet nadir, transfusions, bleeding, thrombosis	Yes
Xu, Song et al, 2018 [64]	China	Phase 3 RCT	rhTPO (77)	15,000 U/day on days 2, 4, 6, and 9	rhIL-11 (31)	NSCLC	Gemcitabine + carboplatin/cisplatin	Peak platelet count, platelet response, platelet nadir, transfusions	Yes
Xu, Jiang et al, 2018 [63]	China	Non-randomized, single-arm clinical trial	rhTPO (198)	300 U/kg/day, adjustments made after platelet count reached $\geq 100 \times 10^9/L$	None (N/A)	Breast cancer	NR	Baseline platelet count, platelet response, transfusions	Yes
Basser et al, 1997 [32]	Australia	Randomized phase 1 trial	MGDF (31)	0.03, 0.1, 0.3, 1.0, 3.0, and 5.0 mg/kg/day, from Day 2 to Day 20 or platelet count $>750 \times 10^9/L$	Placebo (10)	Multiple types of solid tumors	Carboplatin + cyclophosphamide	Baseline platelet count, platelet response, platelet nadir, transfusions, thrombosis, cancer progression	Yes
Fanucchi et al, 1997 [36]	USA	Randomized dose-escalation trial	MGDF (40)	0.03, 0.1, 0.3, 1.0, 3.0, or 5.0 mg/kg/day	Placebo (13)	NSCLC	Carboplatin + paclitaxel	Baseline platelet count, peak platelet count, platelet nadir, thrombosis	Yes

Study Authors, Year	Location	Study Design	Intervention (*N*)	Intervention Dose	Comparison (*N*)	Tumor Type	Chemotherapy	Relevant Outcomes	Meta-analysis (Yes/No)
Archimbaud et al, 1999 [65]	NR	RCT	MGDF (74)	2.5 or 5 μg/kg/day up to 21 doses, single dose, or 7 daily doses	Placebo (34)	AML	Daunorubicin + cytarabine + etoposide	Peak platelet count, platelet response, transfusions, thrombosis	Yes
Basser et al, 2000 [66]	Australia	RCT	MGDF (68)	1, 3, and 10 μg/kg/day before chemotherapy and 5 μg/kg/day after chemotherapy	Other doses in trial (68), Placebo (10)	Multiple types of solid tumors	Carboplatin + cyclophosphamide	Baseline platelet count, peak platelet count, platelet response, grade 3/4 thrombocytopenia, platelet nadir, transfusions, thrombosis	Yes
Schiffer et al, 2000 [69]	USA	RCT	MGDF (38)	2.5 or 5 μg/kg/day	Placebo (19)	AML	Daunorubicin, cytarabine, high dose cytarabine	Baseline platelet count, platelet response, transfusions, thrombosis	Yes
Geissler et al, 2003 [56]	Europe, Australia	RCT	MGDF (69)	30 μg/kg on Day –6, or 30 μg/kg on Day –6 and 10 μg/kg on Days –5 to 6	Placebo (18)	AML	Age < 60 years: cytarabine + mitoxantrone<br>Age ≥ 60 years: daunorubicin + cytarabine + etoposide	Baseline platelet count, platelet response, transfusions, bleeding, thrombosis	Yes
Moskowitz et al, 2007 [51]	USA	Randomized phase 1/2 trial	MGDF (22)	2.5 or 5 μg/kg/day	Placebo (16)	NHL	Ifosfamide, carboplatin, etoposide	Platelet response, grade 3/4 thrombocytopenia, platelet nadir, transfusions, dose delays/reductions	Yes

Study Authors, Year	Location	Study Design	Intervention (*N*)	Intervention Dose	Comparison (*N*)	Tumor Type	Chemotherapy	Relevant Outcomes	Meta-analysis (Yes/No)
**Second-generation thrombopoietic agents**									
Vadhan-Raj et al, 2009 [70]	USA	Phase 1/2 crossover trial	Romiplostim (20)	1, 3, and 10 µg/kg on Days 1 and 3 or 10 µg/kg on Days –5 and 1	Placebo (20)	Solid tumor	Carboplatin + adriamycin and/or ifosfamide	Baseline platelet count, ending platelet count, grade 3-4 thrombocytopenia, platelet nadir, days <100 x 10^9/L, transfusions, dose delays/reductions, bleeding, thrombosis	Yes
Fanale et al, 2009 [55]; NCT00283439 [54]	NR	Phase 1/2 dose- and schedule-finding trial (parallel assignment)	Romiplostim (39)	100, 300, 700, or 1000 µg on Day 1	Other doses in trial (39)	NHL or Hodgkin's lymphoma	Multiple types	Baseline platelet count, grade 3/4 thrombocytopenia, transfusions, bleeding	Yes
Natale et al, 2009 [53]; NCT00413283 [52]	NR	Randomized phase 2 dose- and schedule-finding trial	Romiplostim (51)	250, 500, or 750 µg on Day 2	Placebo (12)	NSCLC	Gemcitabine + carboplatin/cisplatin	Baseline platelet count, ending platelet count, grade 3/4 thrombocytopenia, transfusions, dose delays/reductions, bleeding, thrombosis	Yes
Vadhan-Raj et al, 2010 [62]	USA	Phase 1/2 crossover trial	Romiplostim (24)	1, 3, or 10 µg/kg on Days –5 and 7 or Days 5 and 7	Placebo (12)	NHL	Cyclophosphamide + vincristine + doxorubicin + dexamethasone and rituximab + methotrexate-cytarabine	Platelet nadir, days < 100 x 10^9/L, transfusions, bleeding, thrombosis	Yes

Study Authors, Year	Location	Study Design	Intervention (*N*)	Intervention Dose	Comparison (*N*)	Tumor Type	Chemotherapy	Relevant Outcomes	Meta-analysis (Yes/No)
Parameswaran et al, 2014 [68]	USA	Retrospective case series	Romiplostim (20)	1-2 μg/kg weekly; dose escalated by 1 μg/kg each week until platelet count reached >10 x 10^9/L	None	Multiple types of solid tumors	NR		Yes
Al-Samkari et al, 2021 [12]	USA	Retrospective case series	Romiplostim (173)	Median: 3 μg/kg (IQR 2 μg/kg – 3 μg/kg) weekly	None	Multiple types of solid tumors and hematologic malignancies	Most common: platinum-, gemcitabine-, temozolomide-, and taxane-based regimens	Baseline platelet count, ending platelet count, platelet response, dose delays/reductions, transfusions, % resume chemotherapy, bleeding, thrombosis	Yes
Miao et al, 2018 [67]	USA	Retrospective case series	Romiplostim (42)	Median starting dose: 2 μg/kg	None	Multiple types of solid tumors	Multiple types	Platelet response, thrombosis	Yes
Soff et al, 2019 [50]	USA	Phase 2 randomized, crossover trial	Romiplostim (15)	2 μg/kg weekly, escalated by 1 μg/kg for up to 3 weeks until achieving a platelet count of 100,000/μL	No treatment (8)	Multiple types of solid tumors	Multiple	Baseline platelet count, ending platelet count, platelet response, transfusions, % resume chemotherapy, dose delays/reductions, % switch chemotherapy regimens, thrombosis	Yes

Study Authors, Year	Location	Study Design	Intervention (*N*)	Intervention Dose	Comparison (*N*)	Tumor Type	Chemotherapy	Relevant Outcomes	Meta-analysis (Yes/No)
Ajami et al, 2020 [59]	UK	Retrospective case series	Romiplostim (21)	3 µg/kg weekly	None	Breast cancer	NR	% dose delays/reductions, thrombosis	Yes
**Small-molecule second generation thrombopoietic agents**									
Kellum et al, 2010 [46]	Multicountry	Phase 2 trial	Eltrombopag (134)	50, 75, or 100 mg on Days 2–11 every 3 weeks	Placebo (46)	Multiple types of solid tumors	Carboplatin + paclitaxel	Baseline platelet count, ending platelet count, bleeding, thrombosis	Yes
Chawla et al, 2013 [24]	USA	Phase 1 non-randomized trial	Eltrombopag (12)	75, 100, or 150 mg	No treatment (3)	Soft tissue sarcoma	Doxorubicin + ifosfamide	Baseline platelet count, grade 3/4 thrombocytopenia, bleeding, thrombosis	Yes
Winer et al, 2015 [26]	USA, Europe, India	Phase 1 RCT	Eltrombopag (19)	100, 150, 225, or 300 mg on Days –5 to –1 and 2–6	Placebo (7)	Multiple types of solid tumors	Gemcitabine + cisplatin/carboplatin (I: 9; C: 3); gemcitabine monotherapy (I: 10; C: 4)	Baseline platelet count, peak platelet count, grade 3/4 thrombocytopenia, platelet nadir, dose delays/reductions, thrombosis	Yes
Mukherjee et al, 2016 [77]	NR	Phase 2 trial (non-randomized single-arm)	Eltrombopag (13)	200 mg/day with a maximum one-time dose escalation to 300 mg/day	None (N/A)	AML	Cytarabine + Daunorubicin/ idarubicin	Peak platelet count, platelet response, transfusions	No

Study Authors, Year	Location	Study Design	Intervention (*N*)	Intervention Dose	Comparison (*N*)	Tumor Type	Chemotherapy	Relevant Outcomes	Meta-analysis (Yes/No)
				starting on Day 15					
Strickland et al, 2016 [78]	USA	Phase 1 trial	Eltrombopag (14)	50, 100, 150, 200, and 300 mg; varied dosing schedules	Other doses in trial (14)	AML	Cytarabine	Platelet response	No
Winer et al, 2017 [25]	USA, Europe	Phase 2 RCT	Eltrombopag (52)	100 mg/day for 5 days before and 5 days after chemotherapy	Placebo (23)	Multiple types of solid tumors	Gemcitabine + cisplatin/carboplatin (I: 22; C: 11); gemcitabine monotherapy (I: 30; C: 12)	Ending platelet count, platelet response, grade 3/4 thrombocytopenia, dose delays/reductions, bleeding, thrombosis	Yes
Iuliano et al, 2018 [27]	NR	Non-randomized trial	Eltrombopag (28)	25 mg twice weekly (when platelet counts fell to < 80,000 mm^3)	None	Solid and blood tumors	NR	Peak platelet count, platelet response, platelet nadir	No
Frey et al, 2019 [57]	Multicountry	Phase 2 RCT	Eltrombopag (74)	200 mg daily; 100 mg for patients of East Asian heritage	Placebo (74)	AML	Daunorubicin + cytarabine	Baseline platelet count, platelet response, grade 3/4 thrombocytopenia, transfusions, dose delays/reductions, overall survival, bleeding, thrombosis	Yes
**Mixed thrombopoietic agents**									
Dardis et al, 2017 [1]	USA	Retrospective case series	Eltrombopag (22);	Eltrombopag: 25–200 mg/day;	None (N/A)	Glioma	Temozolomide; after progression:	Platelet response, platelet nadir, % resume	Yes

Study Authors, Year	Location	Study Design	Intervention (*N*)	Intervention Dose	Comparison (*N*)	Tumor Type	Chemotherapy	Relevant Outcomes	Meta-analysis (Yes/No)
			Romiplostim (5)	Romiplostim: 1–10 µg/kg weekly			bevacizumab + lomustine	chemotherapy, thrombosis	

N = number of patients in the study arm. AML, acute myeloid leukemia; MGDF, megakaryocyte growth and development factor; NHL, non-Hodgkin’s lymphoma; N/A, not applicable; NR, not reported; NSCLC, non-small cell lung cancer; RCT, randomized controlled trial; rhIL-11, recombinant human interleukin 11; rhTPO, recombinant human thrombopoietin.
