# Supplementary material for: Systematic literature review and meta-analysis on use of Thrombopoietic agents for chemotherapy-induced thrombocytopenia
Source: PLoS One. 2022 Jun 9;17(6):e0257673. doi: 10.1371/journal.pone.0257673 (PMC9183450; doi:10.1371/journal.pone.0257673)
Supplement: S5 Table — (PDF) [file pone.0257673.s014.pdf]

S5 Table. Baseline characteristics of patient populations in the assessed studies by thrombopoietic agent type and publication year

Study Authors, Year	CIT Intervention and Dose	Sex (M or F)	Median (or Mean) Age, years	Tumor Type	Chemotherapy	Chemotherapy Dose	Median (or Mean) Baseline Platelet Count
**First-generation thrombopoietic agents**							
Vadhan-Raj et al, 1997 [74]	rhTPO given 0.3, 0.6, 1.2, and 2.4 μg/kg of body weight as a single IV dose 3 weeks before chemotherapy start	M: 58.3%	42	Sarcoma	NR	NR	Mean: 597 x 10^9/L
Vadhan-Raj et al, 2000 [35]	rhTPO 0.6, 1.2, 2.4, and 3.6 mg/kg of body weight per day	NR	NR	Gynecologic cancer	Carboplatin	Carboplatin: AUC = 11	Mean: 277 x 10^9/L
Vadhan-Raj et al, 2001 [75]	rhTPO 1.2 μg/kg on Days 1 and 4	NR	59	Ovarian cancer	Carboplatin	Carboplatin: AUC = 11	NR
Vadhan-Raj et al, 2003 [58]	rhTPO 1.2 μg/kg	M: 43.7%	47	Sarcoma	Doxorubicin and ifosfamide	Doxorubicin: 90 mg/m^2 ifosfamide: 10 g/m^2	NR
Bai, Zou et al, 2004 [31]	rhTPO 1.0 μg/kg/day was administered subcutaneously 6–24 hours after the beginning of chemotherapy	NR	NR	Solid tumors: 28.4% Leukemia: 71.6%	NR	NR	NR
Bai, Xu et al, 2004 [48]	rhTPO 1.0 μg/kg/day administered subcutaneously 6 24 hours after chemotherapy	NR	NR	Solid tumors	NR	NR	NR
Dai et al, 2008 [33]	rhTPO 15,000 U/day	NR	NR	Solid tumors	NR	NR	NR

Study Authors, Year	CIT Intervention and Dose	Sex (M or F)	Median (or Mean) Age, years	Tumor Type	Chemotherapy	Chemotherapy Dose	Median (or Mean) Baseline Platelet Count
Yu et al, 2009[61]	rhTPO 15,000 U/day	NR	NR	NSCLC	Gemcitabine and cisplatin	NR	NR
Xu et al, 2011 [49]	rhTPO 300 U/kg/day subcutaneously on Days 2, 4, 6, and 9 after the initiation of chemotherapy	NR	NR	Non-small cell lung cancer	NR	NR	NR
Huang et al, 2014 [34]	TPO injection at Day –4/–2/2–9, (ahead preventive schedule)	M: 63%	Mean 40 ± 16	NHL	Cytarabine	4 g/m^2/cycle	NR
	TPO injection at Days 2–11 (standard preventive cycle)	M: 63%	Mean 40 ± 16	NHL	Cytarabine	4 g/m^2/cycle	NR
Sui et al, 2017 [76]	rhTPO 15,000 U/day starting when platelet count is ≤ 50 x 10^9/L until platelet count increases to > 100 x 10^9/L or for 21 days	NR	NR	AML	Daunorubicin + mitoxantrone + cytarabine	NR	NR
	No treatment	NR	NR	AML	Daunorubicin + mitoxantrone + cytarabine	NR	NR
Wang et al, 2018 [60]	rhTPO10 doses at 15,000 U/dose before chemotherapy (days –4, –2, and 2–9)	M: 65.4%	40	NHL	Cytarabine + cisplatin + dexamethasone (30), cytarabine + ifosfamide + etoposide (11), cytarabine + methotrexate (11)	Cytarabine 2 g/m^2 on day 1 + cisplatin 75 mg/m^2 and dexamethasone OR ifosfamide 7 g/m^2 and etoposide 300 mg/m^2 OR methotrexate 3 g/m^2	NR

Study Authors, Year	CIT Intervention and Dose	Sex (M or F)	Median (or Mean) Age, years	Tumor Type	Chemotherapy	Chemotherapy Dose	Median (or Mean) Baseline Platelet Count
Xu, Song et al, 2018 [64]	rhTPO10 doses at 15,000 U/dose after chemotherapy (days 2-11)	M: 65.4%	40	NHL	Cytarabine + cisplatin + dexamethasone (30), cytarabine + ifosfamide + etoposide (11), cytarabine + methotrexate (11)	Cytarabine 2 g/m^2 on day 1 + cisplatin 75 mg/m^2 and dexamethasone OR ifosfamide 7 g/m^2 and etoposide 300 mg/m^2 OR methotrexate 3 g/m^2	NR
Xu, Song et al, 2018 [64]	rhTPO 15,0000 U on Days 2, 4, 6, and 9	M: 72.7%	Mean 58.7	NSCLC	Gemcitabine/ cisplatin: 49.4%<br>Gemcitabine/ carboplatin: 50.6%	Gemcitabine 1,250 mg/m^2 on Days 1 and 8, + either cisplatin 75 mg/m^2 on Day 1 or divided into 3 days, every 21 days OR carboplatin AUC = 5 mg/mL/min on day 1, every 21 days	NR
Xu, Song et al, 2018 [64]	rhIL-11 3 mg on days 9–15	M: 58.1%	Mean 60.8	NSCLC	Gemcitabine/ cisplatin: 58.1%<br>Gemcitabine/ carboplatin: 41.9%	Gemcitabine 1,250 mg/m^2 on days 1 and 8, + either cisplatin 75 mg/m^2 on day 1 or divided into 3 days, every 21 days OR carboplatin AUC = 5 mg/mL/min on day 1, every 21 days	NR
Xu, Jiang et al, 2018 [63]	rhTPO 300 U/kg daily, adjustments made after platelet count reached ≥100 x 10^9/L	M: 0%	51	Breast cancer	NR	NR	48 x 10^9/L (range: 10–75 x 10^9/L)
Basser et al, 1997 [32]	MGDF 0.03, 0.1, 0.3, 1.0, 3.0, and 5.0 mg/kg/day, from Day 2 by daily subcutaneous injection until platelet	M: 71%	57	NSCLC: 38.7%<br>Carcinoma of unknown primary: 12.9%<br>Gastric cancer: 6.5%	Carboplatin and cyclophosphamide	Carboplatin: 600 mg/m^2 IV<br>Cyclophosphamide: 1200 mg/m^2 IV	273 x 10^9/L (range: 148–487 x 10^9/L)

Study Authors, Year	CIT Intervention and Dose	Sex (M or F)	Median (or Mean) Age, years	Tumor Type	Chemotherapy	Chemotherapy Dose	Median (or Mean) Baseline Platelet Count
	count reached > 750 x 10^9/L or for 20 days			Kidney: 6.5%<br>Small cell lung cancer: 3.2%<br>Other: 32.3%			
Fanucchi et al, 1997 [36]	MGDF 0.03, 0.1, 0.3, 1.0, 3.0, or 5.0 mg/kg/day	M: 68%	58	NSCLC	Carboplatin and paclitaxel	Carboplatin: dose was adjusted on the basis of the measured 24-hour creatinine clearance to give a predicted AUC of the serum concentration plotted against time of 9 mg/mL times the number of minutes.<br>Paclitaxel: 175 mg/mm^2 of body surface area over a 3-hour period	386 x 10^3/mm^3 (range: 184–543 x 10^3/mm^3)
Archimbaud et al, 1999 [65]	MGDF 2.5 or 5 µg/kg/day subcutaneously from 24 hours after the last dose of chemotherapy until a transfusion-independent platelet count ≥ 50 x 10^9/L	M: 52.1%	2.5 µg/kg/day group: 49<br>5 µg/kg/day group: 48	AML	Daunorubicin, cytarabine, and etoposide	Daunorubicin: 45 mg/m^2<br>Cytarabine: 100 mg/m^2<br>Etoposide: 100 mg/m^2	NR
	MGDF 2.5 or 5 µg/kg/day subcutaneously either as a single dose administered on day 7, or for a duration of 7 days (Day 8 to day 14)	M: 61.5%	2.5 µg/kg/day group: 55<br>5 µg/kg/day group: 60				NR

Study Authors, Year	CIT Intervention and Dose	Sex (M or F)	Median (or Mean) Age, years	Tumor Type	Chemotherapy	Chemotherapy Dose	Median (or Mean) Baseline Platelet Count
	MGDF 1 μg/kg/day by subcutaneous injection on Day –14 and 1 day after chemotherapy	M: 91%	59	NSCLC: 36.4%<br><br>Gastric cancer: 18.2%<br><br>Mesothelioma: 9.1%<br><br>Unknown primary: 18.2%<br><br>Other: 18.2%	Carboplatin and cyclophosphamide	Carboplatin: 600 mg/m^2 IV<br>Cyclophosphamide: 1200 mg/m^2 IV	286 x 10^9/L (range: 148–529 x 10^9/L)
Basser et al, 2000 [66]	MGDF 1 μg/kg/day by subcutaneous injection for 3 days (starting on Day –14) and 3 days after chemotherapy	M: 73%	60	NSCLC: 18.2%<br><br>Gastric cancer: 18.2%<br><br>Mesothelioma: 9.1%<br><br>Unknown primary: 18.2%<br><br>Other: 36.4%	Carboplatin and cyclophosphamide	Carboplatin: 600 mg/m^2 IV<br>Cyclophosphamide: 1200 mg/m^2 IV	304 x 10^9/L (range: 149–539 x 10^9/L)
	MGDF 1 μg/kg/day by subcutaneous injection for 7 days (starting on d<br><br>Day –14) and 7 days after chemotherapy	M: 93%	62	NSCLC: 33.3%<br><br>Gastric cancer: 20%<br><br>Mesothelioma: 13.3%<br><br>Unknown primary: 0%<br><br>Other: 33.3%	Carboplatin and cyclophosphamide	Carboplatin: 600 mg/m^2 IV<br>Cyclophosphamide: 1200 mg/m^2 IV	296 x 10^9/L (range: 164–544 x 10^9/L)

Study Authors, Year	CIT Intervention and Dose	Sex (M or F)	Median (or Mean) Age, years	Tumor Type	Chemotherapy	Chemotherapy Dose	Median (or Mean) Baseline Platelet Count
	MGDF 3 µg/kg by subcutaneous injection on Day –11 and 5 µg/kg 3 days after chemotherapy	M: 56%	53	NSCLC: 44.4%<br>Gastric cancer: 0%<br>Mesothelioma: 11.1%<br>Unknown primary: 11.1%<br>Other: 33.3%	Carboplatin and cyclophosphamide	Carboplatin: 600 mg/m^2 IV<br>Cyclophosphamide: 1200 mg/m^2 IV	258 x 10^9/L (range: 191–462 x 10^9/L)
	MGDF 3 µg/kg by subcutaneous injection on Day –7 and 5 µg/kg 3 days after chemotherapy	M: 80%	63	NSCLC: 30%<br>Gastric cancer: 20%<br>Mesothelioma: 20%<br>Unknown primary: 10%<br>Other: 20%	Carboplatin and cyclophosphamide	Carboplatin: 600 mg/m^2 IV<br>Cyclophosphamide: 1200 mg/m^2 IV	303 x 10^9/L (range: 210–601 x 10^9/L)
	MGDF 10 µg/kg by subcutaneous injection on day –7 and 5 µg/kg 3 days after chemotherapy	M: 42%	58	NSCLC: 8.3%<br>Gastric cancer: 8.3%<br>Mesothelioma: 16.7%<br>Unknown primary: 33.3%<br>Other: 33.3%	Carboplatin and cyclophosphamide	Carboplatin: 600 mg/m^2 IV<br>Cyclophosphamide: 1200 mg/m^2 IV	265 x 10^9/L (range: 156–538 x 10^9/L)
Schiffer et al, 2000 [69]	MGDF 2.5 µg/kg/day<br>MGDF 5 µg/kg/day	M: 58%<br>M: 53%	NR<br>NR	AML<br>AML	Daunorubicin, cytarabine, high dose cytarabine	For those aged < 60 years: daunorubicin 45 mg/m^2 IV on Days 1–3	61 x 10^9/L<br>56 x 10^9/L

Study Authors, Year	CIT Intervention and Dose	Sex (M or F)	Median (or Mean) Age, years	Tumor Type	Chemotherapy	Chemotherapy Dose	Median (or Mean) Baseline Platelet Count
					(only for those < 60 years)	cytarabine 100 mg/m^2 continuous infusion Days 1–7<br>high dose cytarabine 2 g/m^2 IV over 90 min 6 doses, every 12 h on Days 8–10<br>(3 cycles) cytarabine 3 g/m^2 over 3 h, 6 doses, every 12 h on Days 1, 3, and 5<br>For those aged ≥ 60 years:<br>daunorubicin 45 mg/m^2 IV on Days 1–3<br>cytarabine 200 mg/m^2 continuous infusion Days 1–7<br>high dose cytarabine 1.5 g/m^2 over 2 h, 8 doses, every 12 h	
Geissler et al, 2003 [56]	MGDF 30 μg/kg single dose on Day –6	M: 57%	51.3	AML	Age < 60 years: cytarabine and mitoxantrone; age ≥ 60 years: daunorubicin, cytarabine, and etoposide	Age < 60 years: cytarabine 1 g/m^2 IV infused every 12 h on Days 1–4, mitoxantrone 12 mg/m^2 IV daily on Days 3–5<br>Age ≥ 60 years: daunorubicin 45 mg/m^2 IV on days 1 and 2, cytarabine 100 mg/m^2 IV every 12 h on Days 1–5, and etoposide 100 mg/m^2 IV on Days 1–5	274 x 10^9/L (Range: 39.9–1420 x 10^9/L)
	MGDF 30 μg/kg administered on Days –5 through day 6	M: 49%	56.1				247 x 10^9/L (range: 13–1311 x 10^9/L)

Study Authors, Year	CIT Intervention and Dose	Sex (M or F)	Median (or Mean) Age, years	Tumor Type	Chemotherapy	Chemotherapy Dose	Median (or Mean) Baseline Platelet Count
Moskowitz et al, 2007 [51]	MGDF 2.5 or 5 μg/kg/day	M: 68.2%	44	NHL	ICE chemotherapy (ifosfamide, carboplatin, etoposide)	(1) Etoposide 100 mg/m^2/day IV on Days 1 to 3; (2) carboplatin administered on Day 2 and dosed to an AUC of 5, calculated using the Calvert formula [creatinine clearance 1 25]; the maximum dose of carboplatin was 800 mg, which corresponds to a creatinine clearance of 135 mg/dL); and (3) ifosfamide 5 g/m^2 mixed with an equal dose of mesna administered via continuous infusion for 24 hours beginning on Day 2	
**Second-generation thrombopoietic agents**							
Fanale et al, 2009 [55]; NCT00283439 [54]	Romiplostim 100 μg subcutaneous injection on the first day after chemotherapy	M: 62.5%	62	Hodgkin's lymphoma or NHL	ICE: 25%<br>RICE: 75%	NR	Mean: 317 x 10^9/L (SD: 162 x 10^9/L)
	Romiplostim 300 μg subcutaneous injection on the first day after chemotherapy	M: 72.7%	58	Hodgkin's lymphoma or NHL	RICE: 82%<br>R-CHOP: 9%<br>ESHAP: 9%	NR	Mean: 294 x 10^9/L (SD: 208 x 10^9/L)
	Romiplostim 500 μg subcutaneous injection	M: 63.6%	55	Hodgkin's lymphoma or NHL	ICE: 18%<br>RICE: 45%<br>R-CHOP: 9%<br>ESHAP: 18%	NR	Mean: 231 x 10^9/L (SD: 79 x 10^9/L)

Study Authors, Year	CIT Intervention and Dose	Sex (M or F)	Median (or Mean) Age, years	Tumor Type	Chemotherapy	Chemotherapy Dose	Median (or Mean) Baseline Platelet Count
	on the first day after chemotherapy				R-ESHAP: 9%		
	Romiplostim 1000 µg subcutaneous injection on the first day after chemotherapy	M: 44.4%	59	Hodgkin's lymphoma or NHL	RICE: 67%<br>R-CHOP: 33%	NR	Mean: 206 x 10^9/L (SD: 78 x 10^9/L)
Natale et al, 2009 [53]; NCT00413283 [52]	Romiplostim 250 µg subcutaneously on Day 2 of each chemotherapy cycle	M: 75%	65	NSCLC	Gemcitabine/ carboplatin: 81%<br>Gemcitabine/ cisplatin: 19%	Standard institutional practice every 21 days	240 x 10^9/L (Range: 111–671 x 10^9/L)
	Romiplostim 500 µg subcutaneously on Day 2 of each chemotherapy cycle	M: 66.7%	65	NSCLC	Gemcitabine/ carboplatin: 56%<br>Gemcitabine/ cisplatin: 44%	Standard institutional practice every 21 days	274 x 10^9/L (range: 100–743 x 10^9/L)
	Romiplostim 750 µg subcutaneously on Day 2 of each chemotherapy cycle	M: 88.2%	64	NSCLC	Gemcitabine/ carboplatin: 59%<br>Gemcitabine/ cisplatin: 35%	Standard institutional practice every 21 days	288 x 10^9/L (range: 115–926 x 10^9/L)
	Placebo	M: 50%	59	NSCLC	Gemcitabine/ carboplatin: 17%<br>Gemcitabine/ cisplatin: 83%	Standard institutional practice every 21 days	317 x 10^9/L (Range: 239–676 x 10^9/L)
Vadhan-Raj et al, 2009 [70]	Romiplostim 1, 3, or 10 µg/kg given subcutaneously as 2 doses given 2 days apart starting from the day after chemotherapy, or days –5 and 1	NR	NR	NR	Carboplatin or adriamycin and/or ifosfamide	Carboplatin: AUC = 11	NR

Study Authors, Year	CIT Intervention and Dose	Sex (M or F)	Median (or Mean) Age, years	Tumor Type	Chemotherapy	Chemotherapy Dose	Median (or Mean) Baseline Platelet Count
Vadhan-Raj et al, 2010 [62]	Romiplostim 1, 3, or 10 µg/kg on Days –5 and 5 or on days 5 and 7	NR	NR	NHL	Cyclophosphamide, vincristine, doxorubicin, and dexamethasone and Rituximab and methotrexate-cytarabine	NR	NR
Parameswaran et al, 2014 [68]	Patients were initiated on approximately 1–2 µg/kg romiplostim subcutaneously, weekly; dose was escalated by approximately 1 µg/kg each week, until platelet count exceeded 100 x 10^9/L	M: 45%	62.5	Colorectal cancer: 10%<br>Cholangio-carcinoma: 25%<br>Anaplastic astrocytoma: 5%<br>Germ cell cancer: 5%<br>Breast cancer: 5%<br>NSCLC: 5%<br>Small cell lung cancer: 5%<br>Ileocecal carcinoma: 5%<br>Pancreatic cancer: 5%<br>Gastro-esophageal: 5%<br>Glioblastoma: 5%<br>Leiomyo-sarcoma: 10%<br>Lymphoma: 5%<br>Neuroendocrine cancer: 5%	NR	NR	56.5 x 10^9/L
Al-Samkari et al, 2021 [12]	Romiplostim 3 µg/kg	M: 55%	60	Breast cancer: 6%<br>Primary CNS lymphoma: 9%	Alkylating agent-based regimen: 6%<br>Antifolate: 1%	NR	Overall cohort: 54 x 10^9/L

Study Authors, Year	CIT Intervention and Dose	Sex (M or F)	Median (or Mean) Age, years	Tumor Type	Chemotherapy	Chemotherapy Dose	Median (or Mean) Baseline Platelet Count
				Colorectal cancer: 13%<br>Gastroesophageal cancer: 10%<br>Genitourinary cancer: 1%<br>Gynecologic cancer: 6%<br>Head and neck cancer: 3%<br>Hepatobiliary cancer: 13%<br>Lung cancer: 8%<br>Lymphoma: 8%<br>Myeloma: 4%<br>Neuroendocrine cancer: 4%<br>Pancreatic cancer: 13%<br>Sarcoma: 3%	Immunomodulatory: 3%<br>FOLFIRI: 5%<br>FOLFIRINOX or FOLFOXIRI: 8%<br>FOLFOX and other platinum/5-FU doublets: 19%<br>Fluoropyrimidine (single agent): 5%<br>Gemcitabine (single agent): 3%<br>Gemcitabine + taxane: 2%<br>Platinum + gemcitabine: 5%<br>Platinum + taxane: 5%<br>Platinum + anthracycline, etoposide, or pemetrexed: 5%<br>Single-agent platinum, anthracycline, or vinca alkaloid: 2%<br>Taxane or taxane-like (single agent): 6%<br>Temozolomide: 8%<br>Targeted therapy: 10%<br>Other: 8%		Solid tumor patients: 60×10^9/L<br><br>Hematologic malignancy patients: 21×10^9/L
Miao et al, 2018 [67]	Romiplostim median average: 2 µg/kg	M: 61.9%	59	Pancreatic cancer: 26.2%<br>Colorectal cancer: 19.0%<br>Cholangiocarcinoma: 11.9%	FOLFOX: 40.5%<br>FOLFIRINOX: 11.9%<br>Carboplatin-containing drug: 11.9%<br>Gemcitabine-containing drug: 11.9%	NR	68×10^9/L

Study Authors, Year	CIT Intervention and Dose	Sex (M or F)	Median (or Mean) Age, years	Tumor Type	Chemotherapy	Chemotherapy Dose	Median (or Mean) Baseline Platelet Count
				Esophageal cancer: 7.1%<br>Gynecologic cancer: 7.1%<br>Breast cancer: 4.8%<br>Lung cancer: 4.8%<br>Hepatocellular carcinoma: 4.8%<br>Other: 14.3%	Investigational drug: 7.1% Other: 16.7%		
Soff et al, 2019 [50]	Romiplostim 1 mg/kg for up to 3 weeks until achieving a platelet count of 100,000/μL	M: 33.3%	50	Breast cancer: 27%<br>Gastrointestinal: 53%<br>Lung cancer: 13%<br>Sarcoma: 7%	Nucleoside analog, carboplatin or cisplatin, an anthracycline, an alkylating agent, or other cytotoxic chemotherapy agent with thrombocytopenia as a known common toxicity	NR	63,000/μL
	No treatment	M: 75%	67	Gastrointestinal cancer: 50%<br>Gynecologic cancer: 13%<br>Lung cancer: 37%	Nucleoside analog, carboplatin or cisplatin, an anthracycline, an alkylating agent, or other cytotoxic chemotherapy agent with thrombocytopenia as a known common toxicity	NR	NR
Ajami et al, 2020 [59]	Romiplostim 3 μg/kg weekly	F: 100%	NR	Breast cancer	NR	NR	NR

Study Authors, Year	CIT Intervention and Dose	Sex (M or F)	Median (or Mean) Age, years	Tumor Type	Chemotherapy	Chemotherapy Dose	Median (or Mean) Baseline Platelet Count
**Small-molecule second generation thrombopoietic agents**							
Kellum et al, 2010 [46]	Eltrombopag 50 mg orally on Days 2–11 every 3 weeks	M: 52%	58.5	NSCLC: 66%<br>Ovarian cancer: 18%<br>Breast cancer: 7%<br>Other: 9%	Carboplatin/ paclitaxel	Carboplatin: AUC 5–6 IV on Day 1 every 3 weeks<br>Paclitaxel: 175–225 mg/m^2 IV on Day 1 every 3 weeks	290.6 Gi/L
	Eltrombopag 75 mg orally on Days 2–11 every 3 weeks	M: 36%	59	NSCLC: 64%<br>Ovarian cancer: 23%<br>Breast cancer: 2%<br>Other:11%	Carboplatin/ paclitaxel	Carboplatin: AUC 5–6 IV Day 1 every 3 weeks<br>Paclitaxel: 175–225 mg/m^2 IV on Day 1 every 3 weeks	317.7 Gi/L
	Eltrombopag 100 mg orally on Days 2–11 every 3 weeks	M: 52%	58	NSCLC: 61%<br>Ovarian cancer: 30%<br>Breast cancer: 2%<br>Other: 7%	Carboplatin/ paclitaxel	Carboplatin: AUC 5–6 IV Day 1 every 3 weeks<br>Paclitaxel: 175–225 mg/m^2 IV on Day 1 every 3 weeks	324.0 Gi/L
Chawla et al, 2013 [24]	Eltrombopag 75 mg for 10 days after chemotherapy or 5 days before and 5 days after chemotherapy	M: 43%	48	Soft tissue sarcoma	Doxorubicin/ Ifosfamide	Doxorubicin: 75 mg/m^2 IV<br>Ifosfamide: 2.5 g/m^2 IV	300/µL (range: 197–368/µL)
	Eltrombopag, 100 mg for 10 days after chemotherapy or 5 days before and 5 days after chemotherapy	M: 50%	30.5	Soft tissue sarcoma	Doxorubicin/ Ifosfamide	Doxorubicin: 75 mg/m^2 IV<br>Ifosfamide: 2.5 g/m^2 IV	264/µL (range: 180–595/µL)
	Eltrombopag 150 mg for 10 days after chemotherapy or 5 days before and 5 days after chemotherapy	M: 0%	59	Soft tissue sarcoma	Doxorubicin/ Ifosfamide	Doxorubicin: 75 mg/m^2 IV<br>Ifosfamide: 2.5 g/m^2 IV	338/µL

Study Authors, Year	CIT Intervention and Dose	Sex (M or F)	Median (or Mean) Age, years	Tumor Type	Chemotherapy	Chemotherapy Dose	Median (or Mean) Baseline Platelet Count
	No treatment	M: 67%	56	Soft tissue sarcoma	Doxorubicin/ ifosfamide	Doxorubicin: 75 mg/m^2 IV Ifosfamide: 2.5 g/m^2 IV	256/μL (range 218–371/μL)
Winer et al, 2015 [26]	Eltrombopag 100, 150, 225, or 300 mg administered on Days –5 to –1 and days 2–6 of each cycle, beginning with cycle 2	M: 22%	53	Bile duct cancer/ cholangiocarcinoma: 33%<br>NSCLC: 11%<br>Breast cancer: 11%<br>Colorectal cancer: 11%<br>Gallbladder cancer: 11%<br>Bladder cancer: 11%<br>Stomach cancer: 11%	Gemcitabine + cisplatin/carboplatin	Gemcitabine 1000–1250 mg/m^2 on Days 1 and 8 and cisplatin 50–80 mg/m^2 on Day 1 or divided on Day 1 and 8, or carboplatin AUC 4–7 on Day 1 of each 21-day cycle	Mean: 108.6 x 10^9/L (SD: 121.8 x 10^9/L)
		M: 70%	69	NSCLC: 40%<br>Breast cancer: 20%<br>Colorectal cancer: 20%<br>Pancreatic cancer: 20%	Gemcitabine monotherapy	1000–1250 mg/m^2 on Days 1, 8, and 15 of a 28-day cycle	Mean: 269.2 x 10^9/L (SD: 184.2 x 10^9/L)
Mukherjee et al, 2016 [77]	Eltrombopag 200 mg/day with a maximum one-time dose escalation to 300 mg/day starting on Day 15	M: 46%	64	AML	Cytarabine + daunorubicin OR idarubicin	Cytarabine 100 mg/m^2 for 7 days + daunorubicin 45 mg/m^2 OR idarubicin 12 mg/m^2 for 3 days	NR
Strickland et al, 2016 [78]	Eltrombopag150 mg starting on Day +3	NR	NR	AML	Cytarabine	Cytarabine 3 g/m^2 (1.5 g/m^2 for patients > 60 years old) IV over 3 hours twice daily on Days 1, 3, and 5	NR

Study Authors, Year	CIT Intervention and Dose	Sex (M or F)	Median (or Mean) Age, years	Tumor Type	Chemotherapy	Chemotherapy Dose	Median (or Mean) Baseline Platelet Count
	Eltrombopag 150 mg starting on Day –1	NR	NR	AML	Cytarabine	Cytarabine 3 g/m^2 (1.5 g/m^2 for patients > 60 years old) IV over 3 hours twice daily on Days 1, 3, and 5	NR
	Eltrombopag 150 mg starting on Day –5	NR	NR	AML	Cytarabine	Cytarabine 3 g/m^2 (1.5 g/m^2 for patients > 60 years old) IV over 3 hours twice daily on Days 1, 3, and 5	NR
	Eltrombopag 200 mg starting on Day –5	NR	NR	AML	Cytarabine	Cytarabine 3 g/m^2 (1.5 g/m^2 for patients > 60 years old) IV over 3 hours twice daily on Days 1, 3, and 5	NR
	Eltrombopag 300 mg starting on Day –5	NR	NR	AML	Cytarabine	Cytarabine 3 g/m^2 (1.5 g/m^2 for patients > 60 years old) IV over 3 hours twice daily on Days 1, 3, and 5	NR
Winer et al, 2017 [25]	Eltrombopag 100 mg/day for 5 days before to and 5 days after chemotherapy	M: 55%	67	Pancreas: 5%<br>Non-small cell lung: 18% Ovary: 18%<br>Bile duct cancer: 14%<br>Bladder cancer: 23%<br>Breast cancer: 5%<br>Gall bladder cancer: 9%<br>Other: 9%	Gemcitabine + carboplatin/ cisplatin	NR	NR
		M: 57%	67.5	Pancreatic cancer: 57%<br>NSCLC: 20%<br>Ovarian cancer: 3%	Gemcitabine	NR	NR

Study Authors, Year	CIT Intervention and Dose	Sex (M or F)	Median (or Mean) Age, years	Tumor Type	Chemotherapy	Chemotherapy Dose	Median (or Mean) Baseline Platelet Count
				Bile duct cancer: 3%<br>Bladder cancer: 3%<br>Breast cancer: 7%<br>Gall bladder cancer: 0%<br>Other: 7%			
		M: 45%	64	Pancreatic cancer: 18%<br>NSCLC: 9%<br>Ovarian cancer: 18%<br>Bile duct cancer: 18%<br>Bladder cancer: 0%<br>Breast cancer: 9%<br>Gall bladder cancer: 9%<br>Other: 18%	Gemcitabine + carboplatin/ cisplatin	NR	NR
	Placebo	M: 42%	66	Pancreatic cancer: 33%<br>NSCLC: 17%<br>Ovarian cancer: 17%<br>Bile duct cancer: 8%<br>Bladder cancer: 0%<br>Breast cancer: 8%<br>Gall bladder cancer: 8%<br>Other: 8%	Gemcitabine	NR	NR
Iuliano et al, 2018 [27]	Eltrombopag 25 mg twice weekly as soon as platelet count falls below 80,000 mm^3	M: 40%	47	Ovarian cancer: 17.9%<br>Colon cancer: 28.6%<br>DLBC lymphoma: 21.4%<br>Breast cancer: 21.4%	NR	NR	-

Study Authors, Year	CIT Intervention and Dose	Sex (M or F)	Median (or Mean) Age, years	Tumor Type	Chemotherapy	Chemotherapy Dose	Median (or Mean) Baseline Platelet Count
				Pancreatic cancer: 10.7%			
Frey, 2019 [57]	Eltrombopag 200 mg daily; 100 mg for patients of East Asian heritage	49%	Mean: 56.7	AML	Daunorubicin + cytarabine	Daunorubicin on Days 1–3 (90 mg/m² for patients aged 18–60 years or 60 mg/m² for patients aged >60 years), cytarabine 100 mg/m^2 on Days 1–7	59.5 x 10^9/L (SD: 43.3 x 10^9/L)
	Placebo	58%	Mean: 56.6	AML	Daunorubicin + cytarabine	Daunorubicin on Days 1–3 (90 mg/m² for patients aged 18–60 years or 60 mg/m² for patients aged > 60 years), cytarabine 100 mg/m^2 on Days 1-7	63.7 x 10^9/L (SD 48.0 x 10^9/L)
**Mixed thrombopoietic agents**							
Dardis et al, 2017 [1]	Eltrombopag 25–200 mg/day	M: 45.5%	58	Glioma	First-line with temozolomide + radiotherapy; after progression: bevacizumab + lomustine	Temozolomide: 75mg/m^2 daily for 6 weeks, then 6–12 cycles for 5/28 days; 1st cycle 150mg/m^2 and then 200mg/m^2 Bevacizumab: 10mg/m^2 every 2 weeks Lomustine: 100mg/m^2 every 6 weeks	NR
	Romiplostim 1–10 µg/kg weekly	M: 0%	63				NR

Chemotherapy: ESHAP, etoposide + solumedrol + high-dose cytarabine + platinum; ICE, ifosfamide + carboplatin + etoposide; RICE, rituximab + ifosfamide + carboplatin + etoposide; R-CHOP, rituximab + cyclophosphamide + doxorubicin + vincristine + prednisone; R-SHAP, rituximab + dexamethasone + cytarabine + cisplatin.

AML, acute myeloid leukemia; AUC, area under the curve; CIT, chemotherapy-induced thrombocytopenia; DLBC, diffuse large B-cell lymphoma; IV, intravenous; MGDF, megakaryocyte growth and development factor; NHL, non-Hodgkin's lymphoma; NR, not reported; NSCLC, non-small cell lung cancer; rhIL-11, recombinant human interleukin-11; rhTPO, recombinant human thrombopoietin; SD, standard deviation.
