# Supplementary material for: Systematic literature review and meta-analysis on use of Thrombopoietic agents for chemotherapy-induced thrombocytopenia
Source: PLoS One. 2022 Jun 9;17(6):e0257673. doi: 10.1371/journal.pone.0257673 (PMC9183450; doi:10.1371/journal.pone.0257673)
Supplement: S6 Table — (PDF) [file pone.0257673.s015.pdf]

S6 Table. Efficacy outcomes by thrombopoietic agent type and publication year

Study Authors, Year	CIT Intervention and Dose	Mean Peak Platelet Count	Median Time to Peak Platelet Count Days	Platelet Count at Nadir	Percentage With Dose-limiting CIT, %	Percentage With Grade 3/4 Thrombocytopenia, % or % [*N1*]	Duration of Grade 3/4 Thrombocytopenia	Percentage Undergoing Transfusions, % or % [*N1*]	Percentage With Chemo-therapy Delays/ Reductions, % or % [*N1*]
**First generation thrombopoietic agents**									
Vadhan-Raj et al, 1997 [74]	TPO given 0.3, 0.6, 1.2, and 2.4 μg/kg of body weight as a single IV dose 3 weeks before chemotherapy start	592 x 10^9/L	NR	NR	NR	NR	NR	NR	NR
Vadhan-Raj et al, 2000 [35]	rhTPO 0.6, 1.2, 2.4, and 3.6 mg/kg of body weight per day	-	15	49 x 10^9/L	NR	NR	NR	26	NR
	No treatment	-	NR	28 x 10^9/L*	NR	NR	NR	59*	NR
Vadhan-Raj et al, 2001 [75]	rhTPO 1.2 μg/kg on Days 1 and 4	Median 2.3-fold	12	NR	NR	NR	NR	NR	NR
Vadhan-Raj et al, 2003 [58]	rhTPO 1.2 μg/kg	NR	NR	71 x 10^3/μL	NR	NR	NR	24.3	3
	No treatment	NR	NR	55 x 10^3/μL	NR	NR	NR	NR	NR
Bai, Zou et al, 2004 [31]	rhTPO 1.0 μg/kg/day was administered subcutaneously 6–24 hours after the beginning of chemotherapy	186 x 10^9/L	NR	13 x 10^9/L	NR	NR	NR	NR	NR
	No treatment	122 x 10^9/L*	NR	12 x 10^9/L*	NR	NR	NR	NR	NR

Study Authors, Year	CIT Intervention and Dose	Mean Peak Platelet Count	Median Time to Peak Platelet Count Days	Platelet Count at Nadir	Percentage With Dose-limiting CIT, %	Percentage With Grade 3/4 Thrombocytopenia, % or % [*N1*]	Duration of Grade 3/4 Thrombocytopenia	Percentage Undergoing Transfusions, % or % [*N1*]	Percentage With Chemo-therapy Delays/ Reductions, % or % [*N1*]
Bai, Xu et al, 2004 [48]	rhTPO 1.0 μg/kg/day administered subcutaneously 6–24 hours after chemotherapy	263.9 x 10^{9}/L (± 142.5)	NR	64.4 x 10^{9}/L (± 45.4)	NR	NR	NR	NR	NR
	No treatment	148.9 x 10^{9}/L (± 67.7)*	NR	52.4 x 10^{9}/L (± 30.9)*	NR	NR	NR	NR	NR
Dai et al, 2008 [33]	rhTPO 15,000 U/day	250.2 x 10^{9}/L (± 156.0)	NR	46.2 x 10^{9}/L (± 20)	NR	NR	NR	11.4 [4]	NR
	rhIL-11a 3 mg/day	160.5 x 10^{9}/L (± 96.4)*	NR	37.2 x 10^{9}/L (± 16.7)*	NR	NR	NR	29.7 [11]	NR
Yu et al, 2009 [61]	rhTPO 15,000 U/day	NR	NR	NR	NR	Grade 3: 53.6 [15]<br>Grade 4: 14.3 [4]	Grade 3: 2.6 days<br>Grade 4: 1 day	NR	NR
	rhIL-11 3 mg/day	NR	NR	NR	NR	Grade 3: 85.3* [29]<br>Grade 4: 41.2* [14]	Grade 3: 4 days*<br>Grade 4: 1.86 days*	NR	NR

Study Authors, Year	CIT Intervention and Dose	Mean Peak Platelet Count	Median Time to Peak Platelet Count Days	Platelet Count at Nadir	Percentage With Dose-limiting CIT, %	Percentage With Grade 3/4 Thrombocytopenia, % or % [*N1*]	Duration of Grade 3/4 Thrombocytopenia	Percentage Undergoing Transfusions, % or % [*N1*]	Percentage With Chemo-therapy Delays/ Reductions, % or % [*N1*]
Xu et al, 2011 [49]	rhTPO 300 U/kg/day subcutaneously on Days 2, 4, 6, and 9 after the initiation of chemotherapy	NR	NR	56 x 10^9/L (± 16)	NR	NR	8 days (± 2)	NR	NR
	No treatment	NR	NR	28 x 10^9/L (± 13)*	NR	NR	12 days (± 3)*	NR	NR
Huang et al, 2014 [34]	TPO injection at Day –-4/–2/2–9, (ahead preventive schedule)	NR	NR	25 x 10^9/L (±10.8)	NR	36.7	3.5 days	NR	NR
	TPO injection at Days 2–11 (standard preventive cycle)	NR	NR	40.8 x 10^9/L (± 15.5)*	NR	13.3	1.2 days*	NR	NR
Sui et al, 2017 [76]	rhTPO 15,000 U/day starting when platelet counts were ≤ 50 x 10^9/L until counts increased to > 100 x 10^9/L or for 21 days	104.63 x 10^9/L	NR	12.43 x 10^9/L	NR	NR	NR	NR	NR
	No treatment	48.86 x 10^9/L	NR	8.28 x 10^9/L	NR	NR	NR	NR	NR
Xu, Song et al, 2018 [64]	rhTPO 15,0000 U on Days 2, 4, 6, and 9	223.5 ±-127.3 x 10^9/L	NR	61.8 ± 39.9 x 10^9/L	NR	NR	NR	4.48	NR

<table>
<thead>
<tr><th>Study Authors, Year</th><th>CIT Intervention and Dose</th><th>Mean Peak Platelet Count</th><th>Median Time to Peak Platelet Count Days</th><th>Platelet Count at Nadir</th><th>Percentage With Dose-limiting CIT, %</th><th>Percentage With Grade 3/4 Thrombocytopenia, % or % [N1]</th><th>Duration of Grade 3/4 Thrombocytopenia</th><th>Percentage Undergoing Transfusions, % or % [N1]</th><th>Percentage With Chemotherapy Delays/ Reductions, % or % [N1]</th></tr>
</thead>
<tbody>
<tr><td rowspan="2">Xu, Jiang et al, 2018 [63]</td><td>rhIL-11 3 mg on Days 9–15</td><td>245.8 ±158.7 x 10^9/L</td><td>NR</td><td>52.8 ± 36.8 x 10^9/L</td><td>NR</td><td>NR</td><td>NR</td><td>7.41</td><td>NR</td></tr>
<tr><td>rhTPO 300 U/kg/day, adjustments made after reaching a platelet count ≥100 x 10^9/L</td><td>NR</td><td>NR</td><td>NR</td><td>NR</td><td>NR</td><td>NR</td><td>14.6</td><td>NR</td></tr>
<tr><td rowspan="2">Wang et al, 2018 [60]</td><td>rhTPO, 10 doses at 15,000 U/dose after chemotherapy (Days–4, –2, and 2–9)</td><td>NR</td><td>NR</td><td>54.67 ± 13.04 x 10^9/L</td><td>NR</td><td>46.2</td><td>1.92 ± 0.63 days</td><td>13.5</td><td>NR</td></tr>
<tr><td>rhTPO10 doses at 15,000 U/dose after chemotherapy (Days 2–11)</td><td>NR</td><td>NR</td><td>46.60 ± 11.10 x 10^9/L</td><td>NR</td><td>67.3</td><td>3.12 ± 0.79 days</td><td>25</td><td>NR</td></tr>
<tr><td rowspan="2">Basser et al, 1997 [32]</td><td>MGDF 0.03, 0.1, 0.3, 1.0, 3.0, and 5.0 mg/kg/day, from Day 2 by daily subcutaneous injection until platelet count reaches > 750 x 10^9/L or 20 days</td><td>NR</td><td>NR</td><td>Median time to nadir 11.8 days</td><td>NR</td><td>NR</td><td>NR</td><td>29</td><td>NR</td></tr>
<tr><td>Placebo</td><td>NR</td><td>NR</td><td>Median time to nadir 14.5 days</td><td>NR</td><td>NR</td><td>NR</td><td>20</td><td>NR</td></tr>
</tbody>
</table>

Study Authors, Year	CIT Intervention and Dose	Mean Peak Platelet Count	Median Time to Peak Platelet Count Days	Platelet Count at Nadir	Percentage With Dose-limiting CIT, %	Percentage With Grade 3/4 Thrombocytopenia, % or % [*N1*]	Duration of Grade 3/4 Thrombocytopenia	Percentage Undergoing Transfusions, % or % [*N1*]	Percentage With Chemo-therapy Delays/ Reductions, % or % [*N1*]
Fanucchi et al, 1997 [36]	MGDF 0.03, 0.1, 0.3, 1.0, 3.0, or 5.0 mg/kg/day	692 x $10^3/mm^3$ (range: 231–1800 x $10^3/mm^3$)	NR	188 x $10^3/mm^3$ (range 68–373 x $10^3/mm^3$) (median 7 days, range 2–16 days)	NR	NR	NR	NR	NR
	Placebo	330 x $10^3/mm^3$ (range: 236–574 x $10^3/mm^3$)	NR	111 x $10^3/mm^3$ (range: 21–307 x $10^3/mm^3$)* (Median: 15 days, range 13–21 days)*	NR	NR	NR	NR	NR
Archimbaud et al, 1999 [65]	MGDF 2.5 or 5 µg/kg/day subcutaneously from 24 hours after the last dose of chemotherapy until a transfusion- independent platelet count of > 50 x 10^9/L. is reached	1,084 x 10^9/L	29	NR	NR	NR	NR	4 days on which a platelet transfusion was required	NR
	MGDF 2.5 or 5 µg/kg/day subcutaneously either as a single dose administered on Day 7, or for a duration of 7 days (Day 8 to Day 14)	517 x 10^9/L	27	NR	NR	NR	NR	5 days on which a platelet transfusion was required	NR

Study Authors, Year	CIT Intervention and Dose	Mean Peak Platelet Count	Median Time to Peak Platelet Count Days	Platelet Count at Nadir	Percentage With Dose-limiting CIT, %	Percentage With Grade 3/4 Thrombocytopenia, % or % [*N1*]	Duration of Grade 3/4 Thrombocytopenia	Percentage Undergoing Transfusions, % or % [*N1*]	Percentage With Chemo-therapy Delays/ Reductions, % or % [*N1*]
	Placebo	390 x 10^9/L	28	NR	NR	NR	NR	5.5 days on which a platelet transfusion was required	NR
Basser et al, 2000 [66]	MGDF 1.0 µg/kg/day alone by subcutaneous injection for 1, 3, or 7 days (starting on Day –14) and either 1, 3, and 7 days after chemotherapy (Part A) or 3 µg/kg given on Day –11 or –7 or 10 µg/kg on Day –7 and 5 µg/kg for 3 days after chemotherapy (Part B)	637 x 10^9/L (range: 244–2260 x 10^9/L)	13 (range 8–17)	27.5 x 10^9/L (range: 4–119 x 10^9/L)	NR	NR	4 days (range: 0–21 days)	NR	NR
Schiffer et al, 2000 [69]	MGDF 2.5 µg/kg/day	NR	NR	NR	NR	NR	NR	NR	NR
	MGDF 5 µg/kg/day	NR	NR	NR	NR	NR	NR	NR	NR
	Placebo	NR	NR	NR	NR	NR	NR	NR	NR
Geissler et al, 2003 [56]	MGDF 30 µg/kg single dose on Day –6	NR	NR	NR	NR	NR	NR	Median 4 days with platelet transfusion	NR
	MGDF 30 µg/kg administered on Days –5 through Day 6	NR	NR	NR	NR	NR	NR	Median 5 days with platelet transfusion	NR

Study Authors, Year	CIT Intervention and Dose	Mean Peak Platelet Count	Median Time to Peak Platelet Count Days	Platelet Count at Nadir	Percentage With Dose-limiting CIT, %	Percentage With Grade 3/4 Thrombocytopenia, % or % [*N1*]	Duration of Grade 3/4 Thrombocytopenia	Percentage Undergoing Transfusions, % or % [*N1*]	Percentage With Chemo-therapy Delays/ Reductions, % or % [*N1*]
	Placebo	NR	NR	NR	NR	NR	NR	Median 4 days with platelet transfusion	NR
	MGDF 2.5 or 5 μg/kg/day	NR	NR	49,000/μL	25	Grade 4: 15	NR	8	25%
Moskowitz et al, 2007 [51]	Placebo	NR	NR	20,000/μL*	58*	Grade 4: 35*	NR	23*	58*
**Second generation thrombopoietic agents**									
	Romiplostim 100 μg subcutaneous injection on the first day after chemotherapy	NR	NR	Change in nadir: 15 x 10^9/L (range: –18–55 x 10^9/L)	NR	62.5	Mean: 3.9 days (SD 1.3)	12.5	NR
Fanale et al, 2009 [55]; NCT00283439 [54]	Romiplostim 300 μg subcutaneous injection on the first day after chemotherapy	NR	NR	Change in nadir: 9 x 10^9/L (range: –10–61 x 10^9/L)	NR	81.8	Mean: 3.6 days (SD: 0.9 days)	0	NR
	Romiplostim 500 μg subcutaneous injection on the first day after chemotherapy	NR	NR	Change in nadir: –4 x 10^9/L (range –16–25 x 10^9/L)	NR	80	Mean: 6 days (SD: 1.5 days)	30	NR

Study Authors, Year	CIT Intervention and Dose	Mean Peak Platelet Count	Median Time to Peak Platelet Count Days	Platelet Count at Nadir	Percentage With Dose-limiting CIT, %	Percentage With Grade 3/4 Thrombocytopenia, % or % [*N1*]	Duration of Grade 3/4 Thrombocytopenia	Percentage Undergoing Transfusions, % or % [*N1*]	Percentage With Chemo-therapy Delays/ Reductions, % or % [*N1*]
	Romiplostim 1000 µg subcutaneous injection on the first day after chemotherapy	NR	NR	Change in nadir: -11 x 10^9/L (range: –29–61 x 10^9/L)	NR	88.9	Mean: 8.3 days (SD: 2.1 days)	33.3	NR
Natale et al, 2009 [53]; NCT00413283 [52]	Romiplostim 250 µg subcutaneously on Day 2 of each chemotherapy cycle	NR	NR	NR	NR	46.7	Mean 3.6 days (SD: 4.3 days)	26.7	27 [4]
	Romiplostim 500 µg subcutaneously on Day 2 of each chemotherapy cycle	NR	NR	NR	NR	38.9	Mean 2.6 days (SD: 3.9 days)	5.6	(22 [4]
	Romiplostim 750 µg subcutaneously on Day 2 of each chemotherapy cycle	NR	NR	NR	NR	43.8	Mean 2.1 days (SD: 2.7 days)	6.3	31 [5]
	Placebo	NR	NR	NR	NR	41.7	Mean 2.1 days (SD: 3.3 days)	8.3	NR
Vadhan-Raj et al, 2009 [70]	Romiplostim 1, 3, or 10 µg/kg given subcutaneously as 2 doses given 2 days apart starting from the day after chemotherapy	NR	NR	(Compared with no treatment nadir) Decreased count: 42.9%	NR	NR	2 days	NR	NR

Study Authors, Year	CIT Intervention and Dose	Mean Peak Platelet Count	Median Time to Peak Platelet Count Days	Platelet Count at Nadir	Percentage With Dose-limiting CIT, %	Percentage With Grade 3/4 Thrombocytopenia, % or % [*N1*]	Duration of Grade 3/4 Thrombocytopenia	Percentage Undergoing Transfusions, % or % [*N1*]	Percentage With Chemo-therapy Delays/ Reductions, % or % [*N1*]
				Stable: 42.9%<br>Increased < 2-fold: 14.3%<br>Decreased count: 16.7%					
	Romiplostim 10 µg/kg given subcutaneously on Days –5 and 1	NR	NR	Stable: 33.3%<br>Increased < 2-fold: 16.7%<br>Increased ≥ 2-fold: 33.3%	NR	NR	2 days	NR	NR
	No treatment	NR	NR	NR	NR	NR	6 days*	NR	NR
Vadhan-Raj et al, 2010 [62]	Romiplostim 1, 3, or 10 µg/kg on Days –5 and 5	NR	NR	24 x 10^9/L (± 5 x 10^9/L)	NR	NR	NR	25	NR
	Romiplostim 1, 3, or 10 µg/kg on Days 5 and 7	NR	NR	16 x 10^9/L (± 4 x 10^9/L)	NR	NR	NR	58	NR
	Placebo	NR	NR	11 x 10^9/L (±1 x 10^9/L)	NR	NR	NR	83	NR
Parameswaran et al, 2014 [68]	Patients were initiated on approximately 1–2 µg/kg romiplostim subcutaneously, weekly; the dose was escalated	NR	NR	NR	100	NR	NR	NR	NR

Study Authors, Year	CIT Intervention and Dose	Mean Peak Platelet Count	Median Time to Peak Platelet Count Days	Platelet Count at Nadir	Percentage With Dose-limiting CIT, %	Percentage With Grade 3/4 Thrombocytopenia, % or % [*N1*]	Duration of Grade 3/4 Thrombocytopenia	Percentage Undergoing Transfusions, % or % [*N1*]	Percentage With Chemo-therapy Delays/ Reductions, % or % [*N1*]
	by approximately 1 µg/kg each week, until platelet count exceeded 100 $x10^9/L$								
Al-Samkari et al, 2021 [12]	Romiplostim 3 µg/kg	NR	NR	NR	NR	NR	NR	Solid tumor patients: 11.3% [17]	24.1 [41]
Miao et al, 2018 [67]	Romiplostim median average: 2.0 µg/kg	NR	NR	NR	NR	NR	NR	-	-
Soff et al, 2019 [50]	Romiplostim 2 µg/kg weekly, increased by 1 µg/kg, for up to 3 weeks until achieving a platelet count of 100,000/µL	NR	NR	Correction of platelet count to > 100,000/µL in 3 weeks: 14/15 (93.3%) patients	NR	NR	NR	0	5.77
	No treatment	NR	NR	Correction of platelet count to > 100,000/µL in 3 weeks: 1/8 (12.5%) patients**	NR	NR	NR	25	NR
Ajami et al (2020) [59]	3 µg/kg weekly	NR	NR	NR	NR	NR	NR	NR	47.6% [10]

Study Authors, Year	CIT Intervention and Dose	Mean Peak Platelet Count	Median Time to Peak Platelet Count Days	Platelet Count at Nadir	Percentage With Dose-limiting CIT, %	Percentage With Grade 3/4 Thrombocytopenia, % or % [*N1*]	Duration of Grade 3/4 Thrombocytopenia	Percentage Undergoing Transfusions, % or % [*N1*]	Percentage With Chemo-therapy Delays/ Reductions, % or % [*N1*]
**Small molecule second generation thrombopoietic agents**									
Kellum et al, 2010 [46]	Eltrombopag 50 mg orally on days 2-11 every 3 weeks	NR	NR	NR	NR	NR	NR	NR	NR
	Eltrombopag 75 mg, orally on Days 2-11, every 3 weeks	NR	NR	NR	NR	NR	NR	NR	NR
	Eltrombopag 100 mg, orally on Days 2-11, every 3 weeks	NR	NR	NR	NR	NR	NR	NR	NR
	Placebo	NR	NR	NR	NR	NR	NR	NR	NR
Chawla et al, 2013 [24]	Eltrombopag 75 mg for 10 days after chemotherapy or 5 days before and 5 days after chemotherapy	NR	NR	NR	NR	43	NR	NR	NR
	Eltrombopag, 100 mg for 10 days after chemotherapy or 5 days before and 5 days after chemotherapy	NR	NR	NR	NR	50	NR	NR	NR
	Eltrombopag 150 mg for 10 days after chemotherapy or 5 days	NR	NR	NR	NR	100	NR	NR	NR

Study Authors, Year	CIT Intervention and Dose	Mean Peak Platelet Count	Median Time to Peak Platelet Count Days	Platelet Count at Nadir	Percentage With Dose-limiting CIT, %	Percentage With Grade 3/4 Thrombocytopenia, % or % [*N1*]	Duration of Grade 3/4 Thrombocytopenia	Percentage Undergoing Transfusions, % or % [*N1*]	Percentage With Chemo-therapy Delays/ Reductions, % or % [*N1*]
	before and 5 days after chemotherapy								
	No treatment	NR	NR	NR	NR	0	NR	NR	NR
Winer et al, 2015 [26]	Eltrombopag 100, 150, 225, or 300 mg administered on Days –5 to –1 and Days 2–6 of each cycle, beginning with cycle 2 (gemcitabine + cisplatin/carboplatin)	NR	NR	Mean: 115 x 10^9/L (SD: 83 x 10^9/L)	NR	33 [3]	NR	NR	22
	Placebo (gemcitabine + cisplatin/carboplatin)	NR	NR	Mean: 53 x 10^9/L (SD: 7 x 10^9/L)	NR	67 [2]	NR	NR	33
	Eltrombopag 100, 150, 225, or 300 mg administered on Days –5 to –1 and Days 2–6 of each cycle, beginning with cycle 2 (gemcitabine monotherapy)	NR	NR	Mean: 143 x 10^9/L (SD: 82 x 10^9/L)	NR	0	NR	NR	40
	Placebo (gemcitabine monotherapy)	NR	NR	Mean: 103 x 10^9/L (SD: 64 x 10^9/L)	NR	25 [1]	NR	NR	75
Mukherjee et al, 2016 [77]	Eltrombopag 200 mg/day with a maximum one-time dose escalation to	719,000/ µL (range 280-	19 (range 13-28)	NR	NR	NR	NR	NR	NR

Study Authors, Year	CIT Intervention and Dose	Mean Peak Platelet Count	Median Time to Peak Platelet Count Days	Platelet Count at Nadir	Percentage With Dose-limiting CIT, %	Percentage With Grade 3/4 Thrombocytopenia, % or % [*N1*]	Duration of Grade 3/4 Thrombocytopenia	Percentage Undergoing Transfusions, % or % [*N1*]	Percentage With Chemotherapy Delays/ Reductions, % or % [*N1*]
	300 mg/day starting on Day 15	1,935,000/ μL)							
	Eltrombopag150 mg starting at Day +3	NR	NR	NR	0	NR	NR	NR	NR
	Eltrombopag 150 mg starting at Day–1	NR	NR	NR	0	NR	NR	NR	NR
Strickland et al, 2016 [78]	Eltrombopag 150 mg starting at Day –5	NR	NR	NR	0	NR	NR	NR	NR
	Eltrombopag 200 mg starting at Day –5	NR	NR	NR	0	NR	NR	NR	NR
	Eltrombopag 300 mg starting at Day –5	NR	NR	NR	0	NR	NR	NR	NR
	Eltrombopag100 mg/day for 5 days before and 5 days after chemotherapy (gemcitabine + carboplatin/cisplatin)	NR	NR	NR	45	77	NR	15.4	77
Winer et al, 2017 [25]	Eltrombopag (gemcitabine)	NR	NR	NR	45	36	NR	15.4	62
	Placebo (gemcitabine + carboplatin/cisplatin)	NR	NR	NR	82	100	NR	13.0	91
	Placebo (gemcitabine)	NR	NR	NR	75	42	NR	13.0	62

Study Authors, Year	CIT Intervention and Dose	Mean Peak Platelet Count	Median Time to Peak Platelet Count Days	Platelet Count at Nadir	Percentage With Dose-limiting CIT, %	Percentage With Grade 3/4 Thrombocytopenia, % or % [*N1*]	Duration of Grade 3/4 Thrombocytopenia	Percentage Undergoing Transfusions, % or % [*N1*]	Percentage With Chemo-therapy Delays/ Reductions, % or % [*N1*]
Iuliano et al, 2018 [27]	Eltrombopag 25 mg twice weekly as soon as platelet count falls below 80,000 mm^3	270,000 mm^3	NR	60,000 mm^3	NR	NR	NR	NR	NR
Frey et al, 2019 [57]	Eltrombopag 200 mg/day; 100 mg for patients of East Asian heritage	NR	NR	NR	NR	5.41	NR	NR	2.7
	Placebo	NR	NR	NR	NR	5.63	NR	NR	4.05
**Mixed thrombopoietic agents**									
Dardis et al, 2017 [1]	Eltrombopag 25–200 mg/day	NR	NR	66.5 (IQR: 43.3–84) x 10^9/L	NR	NR	NR	NR	NR
	Romiplostim 1-10 μg/kg weekly	NR	NR	79.0 (IQR: 61–92) x 10^9/L	NR	NR	NR	NR	NR

N1 = number of patients who experienced the outcome.
*P<0.05 between intervention and control.
**P<0.001 between thrombopoietic agent and control.
CIT, chemotherapy-induced thrombocytopnia; IQR, interquartile range; IV, intravenous; MGDF, megakaryocyte growth and development factor; NR, not reported; rhIL-11, recombinant human interleukin 11; rhTPO, recombinant human thrombopoietin; SD, standard deviation.
