# Supplementary material for: Systematic literature review and meta-analysis on use of Thrombopoietic agents for chemotherapy-induced thrombocytopenia
Source: PLoS One. 2022 Jun 9;17(6):e0257673. doi: 10.1371/journal.pone.0257673 (PMC9183450; doi:10.1371/journal.pone.0257673)
Supplement: S7 Table — (PDF) [file pone.0257673.s016.pdf]

S7 Table. Platelet outcomes for thrombopoietic agent versus control by publication year

Study Authors, Year	CIT Intervention and Dose	Mean Peak Platelet Count	Platelet Count at Nadir
Vadhan-Raj et al, 2000 [35]	rhTPO 0.6, 1.2, 2.4, and 3.6 mg/kg of body weight per day	-	49 x 10^9/L
	No treatment	-	28 x 10^9/L*
Vadhan-Raj et al, 2003 [58]	rhTPO 1.2 µg/kg	NR	71 x 10^3/µL
	No treatment	NR	55 x 10^3/µL
Bai, Zou et al, 2004 [31]	rhTPO 1.0 µg/kg/day was administered subcutaneously 6–24 hours after the beginning of chemotherapy	186 x 10^9/L	13 x 10^9/L
	No treatment	122 x 10^9/L*	12 x 10^9/L*
Bai, Xu et al, 2004 [48]	rhTPO 1.0 µg/kg/day administered subcutaneously 6–24 hours after chemotherapy	263.9 x 10 ^{9}/L (± 142.5)	64.4 x 10^9/L (± 45.4)
	No treatment	148.9 x 10^9/L (± 67.7)*	52.4 x 10^9/L (± 30.9)*
Dai et al, 2008 [33]	rhTPO 15,000 U/day	250.2 x 10^9/L (± 156.0)	46.2 x 10^9/L (± 20)
	rhIL-11a 3 mg/day	160.5 x 10^9/L (± 96.4)*	37.2 x 10^9/L (± 16.7)*
Xu et al, 2011 [49]	rhTPO 300 U/kg/day subcutaneously on Days 2, 4, 6, and 9 after the initiation of chemotherapy	NR	56 x 10^9/L (± 16)
	No treatment	NR	28 x 10^9/L (± 13)*
Sui et al, 2017 [76]	rhTPO 15,000 U/day starting when platelet counts were ≤ 50 x 10^9/L until counts increased to > 100 x 10^9/L or for 21 days	104.63 x 10^9/L	12.43 x 10^9/L
	No treatment	48.86 x 10^9/L	8.28 x 10^9/L
Xu, Song et al, 2018 [64]	rhTPO 15,0000 U on Days 2, 4, 6, and 9	223.5 ±- 127.3 x 10^9/L	61.8 ± 39.9 x 10^9/L
	rhIL-11 3 mg on Days 9–15	245.8 ±158.7 x 10^9/L	52.8 ± 36.8 x 10^9/L

Study Authors, Year	CIT Intervention and Dose	Mean Peak Platelet Count	Platelet Count at Nadir
Basser et al, 1997 [32]	MGDF 0.03, 0.1, 0.3, 1.0, 3.0, and 5.0 mg/kg/day, from Day 2 by daily subcutaneous injection until platelet count reaches > 750 x 10^9/L or 20 days	NR	Median time to nadir 11.8 days
	Placebo	NR	Median time to nadir 14.5 days
Fanucchi et al, 1997 [36]	MGDF 0.03, 0.1, 0.3, 1.0, 3.0, or 5.0 mg/kg/day	692 x 10^3/mm^3 (range: 231–1800 x 10^3/mm^3)	188 x 10^3/mm^3 (range 68–373 x 10^3/mm^3) (median 7 days, range 2–16 days)
	Placebo	330 x 10^3/mm^3 (range: 236–574 x 10^3/mm^3)	111 x 10^3/mm^3 (range: 21–307 x 10^3/mm^3)* (Median: 15 days, range 13–21 days)*
Archimbaud et al, 1999 [65]	MGDF 2.5 or 5 µg/kg/day subcutaneously from 24 hours after the last dose of chemotherapy until a transfusion-independent platelet count of > 50 x 10^9/L. is reached	1,084 x 10^9/L	NR
	MGDF 2.5 or 5 µg/kg/day subcutaneously either as a single dose administered on Day 7, or for a duration of 7 days (Day 8 to Day 14)	517 x 10^9/L	NR
	Placebo	390 x 10^9/L	NR
Moskowitz et al, 2007 [51]	MGDF 2.5 or 5 µg/kg/day	NR	49,000/µL
	Placebo	NR	20,000/µL*
Vadhan-Raj et al, 2009 [70]	Romiplostim 1, 3, or 10 µg/kg given subcutaneously as 2 doses given 2 days apart starting from the day after chemotherapy	NR	(Compared with no treatment nadir)<br>Decreased count: 42.9%<br>Stable: 42.9%<br>Increased < 2-fold: 14.3%

Study Authors, Year	CIT Intervention and Dose	Mean Peak Platelet Count	Platelet Count at Nadir
	Romiplostim 10 µg/kg given subcutaneously on Days –5 and 1	NR	Decreased count: 16.7%<br>Stable: 33.3%<br>Increased < 2-fold: 16.7%<br>Increased ≥ 2-fold: 33.3%
	No treatment	NR	NR
Vadhan-Raj et al, 2010 [62]	Romiplostim 1, 3, or 10 µg/kg on Days –5 and 5	NR	24 x 10^9/L (± 5 x 10^9/L)
	Romiplostim 1, 3, or 10 µg/kg on Days 5 and 7	NR	16 x 10^9/L (± 4 x 10^9/L)
	Placebo	NR	11 x 10^9/L (±1 x 10^9/L)
Soff et al, 2019 [50]	Romiplostim 2 µg/kg weekly, increased by 1 µg/kg, for up to 3 weeks until achieving a platelet count of 100,000/µL	NR	Correction of platelet count to > 100,000/µL in 3 weeks: 14/15 (93.3%) patients
	No treatment	NR	Correction of platelet count to > 100,000/µL in 3 weeks: 1/8 (12.5%) patients**
Winer et al, 2015 [26]	Eltrombopag 100, 150, 225, or 300 mg administered on Days –5 to –1 and Days 2–6 of each cycle, beginning with cycle 2 (gemcitabine + cisplatin/carboplatin)	NR	Mean: 115 x 10^9/L (SD: 83 x 10^9/L)
	Placebo (gemcitabine + cisplatin/carboplatin)	NR	Mean: 53 x 10^9/L (SD: 7 x 10^9/L)
	Eltrombopag 100, 150, 225, or 300 mg administered on Days –5 to –1 and Days 2–6 of each cycle, beginning with cycle 2 (gemcitabine monotherapy)	NR	Mean: 143 x 10^9/L (SD: 82 x 10^9/L)
	Placebo (gemcitabine monotherapy)	NR	Mean: 103 x 10^9/L (SD: 64 x 10^9/L)

N1 = number of patients who experienced the outcome.
*P<0.05 between thrombopoietic agent and control.
**P<0.001 between thrombopoietic agent and control.

CIT, chemotherapy-induced thrombocytopnia; IQR, interquartile range; IV, intravenous; MGDF, megakaryocyte growth and development factor; NR, not reported; rhIL-11, recombinant human interleukin 11; rhTPO, recombinant human thrombopoietin; SD, standard deviation.
