# Supplementary material for: Systematic literature review and meta-analysis on use of Thrombopoietic agents for chemotherapy-induced thrombocytopenia
Source: PLoS One. 2022 Jun 9;17(6):e0257673. doi: 10.1371/journal.pone.0257673 (PMC9183450; doi:10.1371/journal.pone.0257673)
Supplement: S8 Table — (PDF) [file pone.0257673.s017.pdf]

S8 Table. Safety outcomes by thrombopoietic agent type and publication year

Study Authors, Year	CIT Intervention and Dose	Grade ≥2 Bleeding, *N1* (%)	Thrombotic Events, *N1* (%)
**First generation thrombopoietic agents**			
Vadhan-Raj et al, 1997 [74]	rhTPO given 0.3, 0.6, 1.2, and 2.4 μg/kg of body weight as a single intravenous dose 3 weeks before chemotherapy start	NR	NR
Vadhan-Raj et al, 2000 [35]	rhTPO 0.6, 1.2, 2.4, and 3.6 mg/kg/day	Minor bleeding: 1 (3.7)	0
	No treatment	Minor bleeding: 4 (13.8)	0
Vadhan-Raj et al, 2001 [75]	rhTPO 1.2 μg/kg on Days 1 and 4	NR	NR
Vadhan-Raj et al, 2003 [58]	rhTPO 1.2 μg/kg	Minor bleeding: 8 (11.3)<br>Grade 3 bleeding: 2 (2.8)	Thrombosis: 1 (1.4)
	No treatment	Minor bleeding: 0<br>Grade 3 bleeding: 0	Thrombosis: 0 (0)
Bai, Zou et al, 2004 [31]	rhTPO 1.0 μg/kg/day was administered subcutaneously 6–24 hours after the beginning of chemotherapy	NR	NR
	No treatment	NR	NR
Bai, Xu et al, 2004 [48]	rhTPO 1.0 μg/kg/day administered subcutaneously 6–24 hours after chemotherapy	NR	NR
	No treatment	NR	NR
Dai et al, 2008 [33]	rhTPO 15,000 U/day	NR	NR
	rhIL-11a 3 mg/day	NR	NR

Study Authors, Year	CIT Intervention and Dose	Grade ≥2 Bleeding, ***N1*** **(%)**	Thrombotic Events, ***N1*** **(%)**
Sui, et al, 2017 [76]	rhTPO 15,000 U/day starting when platelet count was ≤ 50 x 10^9/L until the count reached >100 x 10^9/L or for 21 days	NR	NR
	No treatment	NR	NR
Wang et al, 2018 [60]	rhTPO10 doses at 15,000 U/dose before chemotherapy (Days –4, –2, and 2–9)	NR	Any thromboembolic events: 0
	rhTPO10 doses at 15,000 U/dose after chemotherapy (Days 2–11)	Intracranial hemorrhage: 1 (1.9)	Central venous catheter thrombosis: 1 (1.9)
Yu et al, 2009 [61]	rhTPO 15,000 U/day	NR	NR
	rhIL-11 3 mg/day	NR	NR
Xu et al, 2011 [49]	rhTPO 300 U/kg/day subcutaneously on Days 2, 4, 6, and 9 after the initiation of chemotherapy	NR	NR
	No treatment	NR	NR
	TPO injection at Day –4/–2/2–9, (ahead preventive schedule)	NR	NR
	TPO injection at Days 2–11 (standard preventive cycle)	NR	NR
Xu, Song et al, 2018 [64]	rhTPO 15,0000 U on Days 2, 4, 6, and 9	NR	NR
	rhIL-11 3 mg on Days 9–15	NR	NR
Xu, Jiang et al, 2018 [63]	rhTPO 300 U/kg/day, adjustments made after reaching platelet count ≥100 x 10^9/L	NR	NR
Basser et al, 1997 [32]	MGDF 0.03, 0.1, 0.3, 1.0, 3.0, and 5.0 mg/kg/day, from Day 2 by daily subcutaneous injection until a platelet count of >750 x 10^9/L or for 20 days	NR	PE: 1 (3.2)<br>Thrombophlebitis: 1 (3.2)

Study Authors, Year	CIT Intervention and Dose	Grade ≥2 Bleeding, *N1* (%)	Thrombotic Events, *N1* (%)
	Placebo	NR	PE: 0<br>Thrombophlebitis: 0
Fanucchi et al, 1997 [36]	MGDF 0.03, 0.1, 0.3, 1.0, 3.0, or 5.0 mg/kg/day	NR	DVT: 1 (2.9)<br>PE: 1 (2.9)<br>Superficial thrombophlebitis: 1 (2.9)
	Placebo	NR	DVT: 0<br>PE: 0<br>Superficial thrombophlebitis: 0
	MGDF 2.5 or 5 µg/kg/day subcutaneously from 24 hours after the last dose of chemotherapy until reaching a transfusion-<br>independent platelet count ≥ 50 x 10^9/L.	NR	Subclavian vein thrombosis (catheter associated): 1 (2.1)<br>Thrombosis in mesenteric vein (operative specimen): 1 (2.1)
Archimbaud et al, 1999 [65]	MGDF 2.5 or 5 µg/kg/day subcutaneously either as a single dose administered on Day 7, or for a duration of 7 days (Day 8 to Day 14)	NR	0
	Placebo	NR	Cerebral infarction: 1 (2.9)<br>Brachiocephalic/subclavian vein thrombosis (catheter associated): 1 (2.9)
Basser et al, 2000 [66]	MGDF 1.0 µg/kg/day alone by subcutaneous injection for 1, 3, or 7 days (starting on Day –14) and either 1, 3, and 7 days after chemotherapy (Part A) or 3 µg/kg given on Day–11 or –7 or 10 µg/kg on Day –7 and 5 µg/kg for 3 days after chemotherapy (Part B)	NR	DVT: 2 (2.94)<br>Peripheral arterial embolism: 1 (1.47)
Schiffer et al, 2000 [69]	MGDF 2.5 µg/kg/day	NR	DVT: 1 (5.3)
	MGDF 5 µg/kg/day	NR	DVT: 1 (5.3)

Study Authors, Year	CIT Intervention and Dose	Grade ≥2 Bleeding, ***N1*** **(%)**	Thrombotic Events, ***N1*** **(%)**
	Placebo	NR	Thrombotic events: 4 (21)
Geissler et al, 2003 [56]	MGDF 30 µg/kg single dose on Day –6	44% patients had ≥ 1 hemorrhagic events	0
	MGDF 30 µg/kg administered on Days –5 through day 6	44% patients had ≥ 1 hemorrhagic events	VTE (bilateral jugular thrombosis): 1 (2.9)<br>Myocardial infarction: 1 (2.9)
	Placebo	50% patients had ≥ 1 hemorrhagic events	Access catheter clotted: 1 (5.6)
Moskowitz et al, 2007 [51]	MGDF 2.5 or 5 µg/kg/day	NR	NR
	Placebo	NR	NR
**Second-generation thrombopoietic agents**			
Fanale et al, 2009 [55];<br>NCT00283439 [54]	Romiplostim 100 µg subcutaneous injection on the first day after chemotherapy	Gastric hemorrhage: 0	Thrombotic events: 0
	Romiplostim 300 µg subcutaneous injection on the first day after chemotherapy	Gastric hemorrhage: 1 (9.1)	Thrombotic events: 0
	Romiplostim 500 µg subcutaneous injection on the first day after chemotherapy	Gastrointestinal hemorrhage: 1 (9.1)<br>Hemorrhoids: 1 (9.1)	Thrombotic events: 0
	Romiplostim 1000 µg subcutaneous injection on the first day after chemotherapy	Gastric hemorrhage: 0	Thrombotic events: 0

Study Authors, Year	CIT Intervention and Dose	Grade ≥2 Bleeding, *N1* (%)	Thrombotic Events, *N1* (%)
		Hemorrhage events: 4 (44)	
Natale et al, 2009 [53]; NCT00413283 [52]	Romiplostim 250 μg subcutaneously on Day 2 of each chemotherapy cycle	Hemorrhage: 1 (6.25)	Thrombophlebitis: 1 (6.25)<br>PE: 0 (0%)
	Romiplostim 500 μg subcutaneously on Day 2 of each chemotherapy cycle	Hemorrhage: 0	Thrombophlebitis: 0<br>PE: 0 (0%)<br>Thrombotic event: 1 (6.25)
	Romiplostim 750 μg subcutaneously on Day 2 of each chemotherapy cycle	Hemorrhage: 0	Thrombophlebitis: 0<br>PE: 1 (6.25%)
	Placebo	Hemorrhage: 0	Thrombophlebitis: 0<br>PE: 0
Vadhan-Raj et al, 2009 [70]	Romiplostim 1, 3, or 10 μg/kg given subcutaneously as 2 doses given 2 days apart starting from the day after chemotherapy	NR	PE: 1 (7.14)
	Romiplostim 10 μg/kg given subcutaneously on Days –5 and 1	NR	PE: 1 (7.14)
	No treatment	NR	NR
Vadhan-Raj et al, 2010 [62]	Romiplostim 1, 3, or 10 μg/kg on Days –5 and 5	NR	VTE: 4 (11.1)<br>DVT: 2 (5.6)<br>PE: 2 (5.6)
	Romiplostim 1, 3, or 10 μg/kg on Days 5 and 7	NR	VTE: 4 (11.1)<br>DVT: 2 (5.6)

Study Authors, Year	CIT Intervention and Dose	Grade ≥2 Bleeding, *N1* (%)	Thrombotic Events, *N1* (%)
			PE: 2 (5.6)
	Placebo	NR	DVT: 3 (33.3)<br>PE: 2 (22.2)
	No treatment	NR	NR
Parameswaran et al, 2014 [68]	Patients were initiated on approximately 1–2 µg/kg romiplostim subcutaneously, weekly. The dose was escalated by approximately 1 µg/kg each week, until platelet count exceeded 100 x10^9/L	NR	3 (15)
Al-Samkari et al, 2021 [12]	Romiplostim 3 µg/kg	11 (6.4)	Thrombotic event: 8 (4.6)<br>DVT: 4 (2.3)<br>PE: 2 (1.2)<br>Renal vein thrombosis: 1 (0.6)<br>Central venous catheter-associated thrombosis: 1
Miao et al, 2018 [67]	Romiplostim median average: 2 µg/kg	NR	Any thromboembolic event: 6 (14.3)<br>PE: 3 (7.1)<br>DVT: 3 (7.1)
Soff et al, 2019 [50]	Romiplostim 2 mg/kg weekly, escalated by 1 mg/kg for up to 3 weeks until achieving a platelet count of 100,000/µL	NR	Any thromboembolic events: 6 (10.2)<br>PE: 2 (3.4)<br>Proximal DVT: 2 (3.4)<br>Calf vein DVT: 2 (3.4)

Study Authors, Year	CIT Intervention and Dose	Grade ≥2 Bleeding, *N1* (%)	Thrombotic Events, *N1* (%)
	No treatment	NR	NR
Ajami et al, 2020 [59]	Romiplostim 3 µg/kg weekly	NR	Thrombotic event: 0
**Small-molecule second generation thrombopoietic agents**			
Kellum et al, 2010 [46]	Eltrombopag 50 mg orally on days 2-11 every 3 weeks	Any bleeding events: 4 (9)<br>Grade ≥3 bleeding: 1 (2)	Any thromboembolic events: 2 (5)<br>Grade ≥3: 1 (2)
	Eltrombopag 75 mg orally on days 2-11 every 3 weeks	Any bleeding events: 1 (2)<br>Grade ≥3 bleeding: 1 (2)	Any thromboembolic events: 2 (5)<br>Grade ≥3: 2 (5)
	Eltrombopag 100 mg orally on days 2-11 every 3 weeks	Any bleeding events: 3 (7)<br>Grade ≥3 bleeding: 0	Any thromboembolic events: 6 (13)<br>Grade ≥3: 5 (11)
	Placebo	Any bleeding events: 4 (9)<br>Grade ≥3 bleeding: 1 (2)	Any thromboembolic events: 3 (7)<br>Grade ≥3: 3 (7)
Chawla et al, 2013 [24]	Eltrombopag 75 mg for 10 days after chemotherapy or 5 days before and 5 days after chemotherapy	NR	PE: 0<br>Subclavian vein thrombosis: 2 (28.6)
	Eltrombopag, 100 mg for 10 days after chemotherapy or 5 days before and 5 days after chemotherapy	NR	PE: 0 (0%)<br>Subclavian vein thrombosis: 0
	Eltrombopag 150 mg for 10 days after chemotherapy or 5 days before and 5 days after chemotherapy	Grade 3 epistaxis: 1 (100)	PE: 0<br>Subclavian vein thrombosis: 0
	No treatment	Grade 2 hematemesis: 1 (33.3)<br>Grade 2 hematuria: 1 (33.3)	PE:: 1 (33)<br>Subclavian vein thrombosis: 0

Study Authors, Year	CIT Intervention and Dose	Grade ≥2 Bleeding, *N1* (%)	Thrombotic Events, *N1* (%)
		Grade 2 hemoptysis: 1 (33.3)	
Winer et al, 2015 [26]	Eltrombopag 100, 150, 225, or 300 mg administered on Days –5 to –1 and Days 2–6 of each cycle, beginning with cycle 2 (gemcitabine + cisplatin/carboplatin)	NR	22% patients had DVT/VTE
	Placebo (gemcitabine + cisplatin/carboplatin)	NR	0 patients had DVT/VTE
	Eltrombopag 100, 150, 225, or 300 mg administered on Days –5 to –1 and Days 2–6 of each cycle, beginning with cycle 2 (gemcitabine monotherapy)	NR	10% patients had DVT/VTE
	Placebo (gemcitabine monotherapy)	NR	0 patients had DVT/VTE
Mukherjee et al, 2016 [77]	Eltrombopag 200 mg/day with a maximum one-time dose escalation to 300 mg/day starting on Day 15	NR	NR
Strickland et al, 2016 [78]	Eltrombopag150 mg starting on Day +3	NR	NR
	Eltrombopag 150 mg starting on Day –1	NR	NR
	Eltrombopag 150 mg starting on Day –5	NR	NR
	Eltrombopag 200 mg starting on Day –5	NR	NR
	Eltrombopag 300 mg starting on Day –5	NR	NR
Winer et al, 2017 [25]	Eltrombopag100 mg/d for 5 days prior to and 5 days post chemotherapy (gemcitabine + carboplatin/cisplatin)	Grade 1/2 bleeding: 2 (9)	Any thromboembolic event: 1 (5)
	Eltrombopag (gemcitabine)	Grade 1/2 bleeding: 1 (3)	Any thromboembolic event: 4 (13)<br>Myocardial infarction: 1 (3.3)

Study Authors, Year	CIT Intervention and Dose	Grade ≥2 Bleeding, *N1* (%)	Thrombotic Events, *N1* (%)
			PE: 1 (3.3)<br>DVT: 2 (6.7)
	Placebo (Gemcitabine + carboplatin/cisplatin)	Grade 1/2 bleeding: 5 (45)	Any thromboembolic event: 1 (9)
	Placebo (Gemcitabine)	Grade 1/2 bleeding: 0 (0)	Any thromboembolic event: 1 (8)<br>Portal vein thrombosis: 1 (8)
Iuliano et al, 2018 [27]	Eltrombopag 25 mg twice weekly as soon as platelet count falls below 80,000 mm^3	NR	NR
Frey et al, 2019 [57]	Eltrombopag 200 mg/day; 100 mg for patients of East Asian heritage	Grade 3–5 bleeding: 7 (9.46)	Any thromboembolic events: 5 (6.76)<br>Cerebrovascular accident: 1 (1.35)<br>Myocardial infarction: 1 (1.35)
	Placebo	Grade 3–5 bleeding: 1 (1.41)	Any thromboembolic events: 4 (5.63)
**Mixed thrombopoietic agents**			
Dardis et al, 2017 [1]	Eltrombopag 25-200 mg/day	NR	PE: 1 (4.5)
	Romiplostim1-10 µg/kg weekly	NR	NR

N1 = number of patients who experienced the outcome.
AML, acute myeloid leukemia; CIT, chemotherapy-induced thrombocytopenia; DVT, deep venous thrombosis; MGDF, megakaryocyte growth and development factor; NHL, non-Hodgkin's lymphoma; NR, not reported; NSCLC, non-small cell lung cancer; PE, pulmonary embolism; PO, orally; RCT, randomized controlled trial; rhIL-11, recombinant human interleukin 11; rhTPO, recombinant human thrombopoietin; QW, weekly; Q3W, every 3 weeks; SD, standard deviation; VTE, venous thromboembolism.
