# Supplementary material for: Systematic literature review and meta-analysis on use of Thrombopoietic agents for chemotherapy-induced thrombocytopenia
Source: PLoS One. 2022 Jun 9;17(6):e0257673. doi: 10.1371/journal.pone.0257673 (PMC9183450; doi:10.1371/journal.pone.0257673)
Supplement: S9 Table — (PDF) [file pone.0257673.s018.pdf]

S9 Table. Sensitivity analyses including studies with thrombopoietic agent/comparator pairs only

	Rate in Intervention Group[a]			Rate in Comparison Group[a]			*P*-value
**Analysis**	***n***	**Rate (95% CI)**	***P*-Het; I^2**	***n***	**Rate (95% CI)**	***P*-Het; I^2**	
Chemotherapy dose delay/reduction	4	26.0% (6.5-64.0%)	<0.001; 92.01%	4	40.4% (9.6-81.2%)	<0.001; 91.0%	0.601
Grade 3/4 thrombocytopenia	6	26.5% (12.4-47.8%)	<0.001; 86.1%	6	34.8% (13.0-65.7%)	<0.001; 82.7%	0.625
Platelet transfusion	7	21.4% (13.6-31.8%)	0.054; 51.6%	7	31.7% (14.8-55.4%)	0.001; 74.6%	0.350
Grade ≥ 2 bleeding	8	6.5% (1.4-25.2%)	<0.001; 93.5%	8	16.5% (5.1-42.2%)	<0.001; 75.3%	0.319
Thrombosis (any)[b]	14	7.7% (5.8-10.2%)	0.702; 0.0%	14	12.5% (6.8-21.8%)	0.121; 31.8%	0.154
Deep venous thrombosis	3	7.6% (2.8-19.1%)	0.233; 31.4%	3	33.3% (11.1-66.7%)	0.998; 0.0%	0.042
Pulmonary embolism	5	3.5% (1.5-8.2%)	0.933; 0.0%	5	25.3% (8.3-55.7%)	0.997; 0.0%	0.006
Thrombophlebitis	3	2.6% (0.08-7.8%)	0.933; 0.0%	3	3.6% (0.2-38.4%)	0.999; 0.0%	0.838
Other[c]	4	5.1% (2.0-12.7%)	0.181; 38.5%	4	5.4% (2.0-13.5%)	0.995; 0.0%	0.940

n = number of studies with a study arm reporting the endpoint of interest.
[a]The rate in comparison group for each thrombopoietic agent is the meta-analysis for the comparison groups in studies that evaluated each thrombopoietic agent only. For example, for eltrombopag, the rate in the intervention group is the rate among all studies with an eltrombopag arm; the rate in the comparison group is the rate among those eltrombopag studies but only in the comparison arm, it does not include comparison arms of studies evaluating other thrombopoietic agents.
[b]The overall measure of thrombosis reported in studies. Subgroups do not add up to 51 as some studies reported multiple types of thrombotic events, which were included as a summary measure in the overall thrombosis analysis.
[c]Specific types of thrombotic events reported in < 3 studies (insufficient number for a meta-analysis); includes subclavian vein thrombosis, central venous catheter thrombosis, portal vein thrombosis, renal vein thrombosis, myocardial infarction, and cerebrovascular accident.
CI, confidence interval; Het, heterogeneity; I^2, degree of heterogeneity; MGDF, megakaryocyte growth and development factor; N/A, not applicable; rhTPO, recombinant human thrombopoietin.
