# Supplementary material for: Systematic literature review and meta-analysis on use of Thrombopoietic agents for chemotherapy-induced thrombocytopenia
Source: PLoS One. 2022 Jun 9;17(6):e0257673. doi: 10.1371/journal.pone.0257673 (PMC9183450; doi:10.1371/journal.pone.0257673)
Supplement: S1 Results — (PDF) [file pone.0257673.s019.pdf]

S1 Results. Study characteristics and designs, thrombopoietic agent doses, and baseline demographics of studies that met the eligibility criteria for assessment

Study characteristics

Detailed characteristics of the 39 studies that met the eligibility criteria for assessment are presented in S4 Table for first generation thrombopoietic agents including recombinant human thrombopoietin (rhTPO) and megakaryocyte growth and development factor (MGDF), second generation thrombopoietic agent (romiplostim) and small-molecule second generation thrombopoietic agents (eltrombopag, avatrombopag, and lusutrombopag), mixed thrombopoietic agents, and unspecified thrombopoietic agent. The assessment included a total of 2404 patients. Studies were conducted in the United States, China, Australia, and across multiple regions, including Europe and India. Fourteen of the studies evaluated rhTPO [31, 33-35, 48, 49, 58, 60, 61, 63, 74-76], 7 MGDF [32, 36, 51, 56, 65, 66, 69], 9 romiplostim [50, 53, 55, 59, 62, 67, 68, 70], and 8 eltrombopag [24-27, 46, 57, 77, 78]. One case series [1] evaluated CIT in patients who had received either romiplostim or eltrombopag. In a retrospective study evaluating the incidence, clinical outcomes, and cost of chemotherapy-induced thrombocytopenia (CIT) in US clinical practice [73], the administered thrombopoietic agents, if any, were not identified; however, the drug code for thrombopoietic agents was one of the criteria used to identify CIT. No studies evaluating avatrombopag or lusutrombopag and no studies evaluating the cost-effectiveness of thrombopoietic agents in preventing or treating CIT met the eligibility criteria for inclusion in this analysis. Twenty-five of the studies evaluated thrombopoietic agents for preventing and 13 for treating CIT.

Study designs

Of the 39 studies, 5 were retrospective case series reviews [1, 12, 59, 67, 68], 14 were non-randomized controlled trials, and the remaining 20 were randomized controlled trials (Table 1,

S4 Table). Nine utilized a crossover design in the same patient population [31, 34, 48-50, 58, 60, 62, 70] (S4 Table). Thirty studies (a total of 1973 patients) [1, 12, 24-26, 32-36, 46, 50, 51, 53, 55-63, 65-70] reported data for outcomes in ≥ 3 studies, including the outcomes of chemotherapy dose delays and/or reductions, grade 3/4 thrombocytopenia, platelet transfusions, grade ≥ 2 bleeding, and thrombosis, and data from these studies were meta-analyzed to evaluate thrombopoietic agent versus a control (comparator, placebo, or no treatment) in the prevention or treatment of CIT (S4 Table).

Thrombopoietic agent doses and dosing schedules

The thrombopoietic agent doses and dosing schedules varied across the 39 assessed studies (S4 Table).

rhTPO was given at a dose of 15,000 units/day either before or after chemotherapy [33, 60, 61]; 15,000 units/day starting when platelet counts were ≤ 50 x 10^9/L until > 100 x 10^9/L or for 21 days [76]; 15,000 units/day on days 2, 4, 6, 9 [63]; 1 µg/kg/day 6–24 hours after chemotherapy [31, 48]; 300 units/kg/day, daily or on days 2, 4, 6, and 9 after chemotherapy initiation [49, 63]; 1.2 µg/kg and on days 1 and 4 [75]; and varying doses of 1.2 µg/kg, 2.4 µg/kg, or 3.6 µg/kg [58], 0.6– 3.6 mg/kg/day [35]; 0.3–2.4 µg/kg [74]; or as an injection on days –4/–2/2–9, (ahead preventive schedule) or as an injection on days 2–11 (standard preventive cycle) [34].

MGDF was given at a dose of 2.5–5 µg/kg/day [51, 65, 69]; 0.03–5 mg/kg/day until platelet counts increased to > 750 x 10^9/L [32]; 30 µg/kg given as a single dose on day –6 before chemotherapy, or 30 µg/kg on Day –6 and 10 µg/kg on days –5 through day 6 of chemotherapy [56]; 1 µg/kg/day, 3 µg/kg/day, or 10 µg/kg/day before chemotherapy and 5 µg/kg/day after chemotherapy [66]; or between 0.03 mg/kg/day and 5 mg/kg/day [36].

Romiplostim doses given varied between 1, 3, and 10 µg/kg administered subcutaneously before and after chemotherapy [62, 70]; median starting dose of 2 µg/kg weekly

[67]; 100-1000 μg given once on either Day 1 or Day 2 after chemotherapy initiation [52-55]; and median 3 μg/kg body weight weekly [12, 59]. The other two studies include dose escalations, with one study starting romiplostim at 2 μg/kg weekly and escalating the dose by 1 μg/kg for up to 3 weeks until achieving a platelet count of 100,000/μL [50] and starting dose of 1 μg/kg to 2 μg/kg weekly with dose escalations by 1 μg/kg each week until until platelet count exceeded 100 x 10^9/L [68].

Eltrombopag studies also reported different doses and schedules of the drug. Winer et al 2015 [26] administered four dose cohorts of eltrombopag 100 mg, 150 mg, 225 mg, or 300 mg given daily from days –5 to –1 and 2-6 of each chemotherapy cycle. Strickland et al 2016 [78] administered eltrombopag 50, 100, 150, 200, or 300 mg at various dosing schedules. Chawla et al 2013 [24] administered eltrombopag 75, 100, or 150 mg either for ten days after chemotherapy or 5 days before and 5 days after chemotherapy. Iuliano et al 2018 [27] administered eltrombopag 25 mg twice weekly when platelet counts dipped below 80,000 mm^3, and Kellum et al 2010 [46] administered eltrombopag doses of 50 mg, 75 mg, and 100 mg given on days 2-11 of the chemotherapy cycle every 3 weeks. Mukherjee et al 2016 [77] administered eltrombopag 200 mg/day with a maximum one-time dose escalation to 300 mg/day starting on day 15. Winer et al 2017 [25] administered eltrombopag 100 mg/day for 5 days before and after chemotherapy was started. Frey et al 2019 [57] administered eltrombopag 200 mg/day, with 100 mg/day for patients of East Asian heritage.

Baseline demographics of patient populations in the assessed studies

Baseline characteristics of patient populations as reported in the 39 assessed studies are summarized in S5 Table. The proportion of male patients ranged from 0% in each arm of two studies [24, 59] to 93% in an arm of another study [66]. The reported median ages were mostly between 40 and 60 years, with 8 studies reporting median ages of > 60 years. The most common cancers reported in the studies were hematopoietic malignancies (*n* = 12 studies;

30.8% of studies) and non-small cell lung cancer (NSCLC) (*n* = 5; 12.8%), and many studies (*n* = 16; 41.0%) reported a mixture of cancers in their patient populations. Platinum-based treatments (*n* = 18; 46.2%) and cytarabine (*n* = 11; 28.2%) were the most commonly reported chemotherapy types.

As the thrombopoietic agent was given either as treatment for CIT or prophylactically, some patient populations did not have thrombocytopenia (defined as platelet counts < 50 x 10^9/L for this analysis) at baseline. The median or mean platelet count at baseline ranged from 48 x 10^9/L [63] to 597 x 10^9/L [74]. Eighteen studies evaluated thrombopoietic agent intervention as treatment for CIT (median or mean baseline platelet count ranged from 48 x 10^9/L to 324 x 10^9/L) [1, 12, 31, 33, 46, 48, 50, 52-55, 59, 61, 63, 67-69, 76-78]. The remaining 21 studies evaluated thrombopoietic agent to prevent CIT (median or mean baseline platelet count ranged from 59.5 x 10^9/L to 597 x 10^9/L).
