# Supplementary material for: Systematic literature review and meta-analysis on use of Thrombopoietic agents for chemotherapy-induced thrombocytopenia
Source: PLoS One. 2022 Jun 9;17(6):e0257673. doi: 10.1371/journal.pone.0257673 (PMC9183450; doi:10.1371/journal.pone.0257673)
Supplement: S2 Results — (PDF) [file pone.0257673.s020.pdf]

S2 Results. Efficacy and safety outcomes

Efficacy outcomes for the 39 studies that met the eligibility criteria for assessment

In the 39 studies that met the eligibility criteria for data extraction, mean peak platelet count ranged from 48.86 x 10^9/L with no treatment [76] and 104.63 x 10^9/L with rhTPO [76] to 1,084 x 10^9/L with MGDF [65] (S6 Table). The median time to reach peak platelet count ranged from 12 days [75] and 15 days [35] with rhTPO to 27 days and 29 days with MGDF [65]. Platelet count at nadir varied widely across studies as well, ranging from 8.29 x 10^9/L with a no-treatment group cycle [76] and 12.43 x 10^9/L with rhTPO [76] to 188 x 10^9/L in an MGDF group cycle [36].

Moskowitz et al 2007 [51] reported dose-limiting CIT in 58% of patients in the placebo group, which was reduced to 25% in patients who received MGDF. Across other studies, dose-limiting CIT varied from 0% in all arms of Strickland et al 2016 [78], to 100% in patients receiving romiplostim in Parameswaran et al 2014 [68]. The proportion of patients experiencing grade 3/4 thrombocytopenia ranged from 0% in the no treatment group of Chawla et al 2013 [24] to 100% in the eltrombopag 150 mg group of Chawla et al 2013 [24] and the gemcitabine + carboplatin + cisplatin group of Winer et al 2017 [25]. The mean duration of grade 3/4 thrombocytopenia ranged from 1 day in the rhTPO arm of Yu et al 2009 [61], and 8 days in the rhTPO arm of Xu et al 2011 [49] to 12 days in the no-treatment arm of Xu et al 2011 [49]. The proportion of patients undergoing transfusions ranged from 0% in the romiplostim 300 µg group in Fanale et al 2009 [54, 55] to 83% in the placebo group in Vadhan-Raj et al 2010 [62].

Chemotherapy dose delays or dose reductions occurred in 22% of patients in the eltrombopag group receiving gemcitabine + cisplatin/carboplatin and 40% in the eltrombopag group receiving gemcitabine monotherapy in Winer et al 2015 [26]. Vadhan-Raj et al 2003 [58] reported 3% and Moskowitz et al 2007 [51] reported 25% of patient populations experiencing chemotherapy delay and/or dose reduction. Al-Samkari et al 2021 [12] reported 24.1% of patients experiencing dose delays or reductions. Ajami et al 2020 [59] reported 47.6% of

patients had dose delays or reductions. Natale et al 2009 [52, 53] reported dose delays and/or reductions in 22% to 31% of patients across treatment groups.

Efficacy outcomes comparing a thrombopoietic agent study arm with a control (comparator, placebo, or no treatment) arm

Efficacy outcomes were compared between the thrombopoietic agent study arms of assessed studies with the control (comparator, placebo, or no treatment) arms. Studies comparing rhTPO with no treatment reported significantly higher mean peak platelet count [32], higher platelet counts at nadir [31, 33-35], shorter duration of thrombocytopenia [49], and fewer transfusions [35] with rhTPO (S6 Table). Three studies compared recombinant human interleukin 11 (rhTPO with rhIL-11) as a comparator [33, 61, 63]; of these, one study [61] reported significantly decreased rates and duration of grade 3/4 thrombocytopenia with rhTPO. MGDF was reported to significantly increase platelet counts at nadir [36], reduce the proportion of patients with dose-limiting CIT and dose delays and/or reductions, reduce the proportion of patients with grade 3/4 thrombocytopenia, and reduce the number of patients requiring transfusions [51]. Among studies comparing romiplostim with placebo or no treatment, romiplostim was found to significantly decrease the duration of grade 3/4 thrombocytopenia [70], increase the proportion of patients with platelet correction within 3 weeks [50], and decrease the proportion of patients needing transfusions [62]. Eltrombopag was reported to increase platelet count [46], shorten time to recovery from platelet nadir [25], and reduce chemotherapy dose delays or reductions [25, 26].

Safety outcomes

Safety outcomes reported in the 39 assessed studies are summarized in S7 Table. A number of the studies reported bleeding events (11 of 39 studies; 28.2%), ranging from 0% of patients in 2 studies [52-55] to 44% of patients experiencing ≥ 1 hemorrhagic events in another study [56]. Most studies reported thrombotic events (23 of 39 studies; 59.0%), with DVT and thrombophlebitis being the most common thrombotic events. Cancer progression was reported

in only one study [32], with 48% of patients in that study experiencing disease progression. Survival was rarely reported in the studies (*n* = 2; 5.1%) [51, 69]. One study reported a median overall survival time of 12.3 months [69]. Another study reported that after a median follow-up of 8.5 years, the overall survival rate was 59% and the event-free survival rate was 36% [51]. One study reported a measure of quality of life [52, 53]; one patient (2%) in that study experienced depression as an adverse event.
