# Supplementary material for: Systematic literature review and meta-analysis on use of Thrombopoietic agents for chemotherapy-induced thrombocytopenia
Source: PLoS One. 2022 Jun 9;17(6):e0257673. doi: 10.1371/journal.pone.0257673 (PMC9183450; doi:10.1371/journal.pone.0257673)
Supplement: S1 Fig — (PDF) [file pone.0257673.s002.pdf]

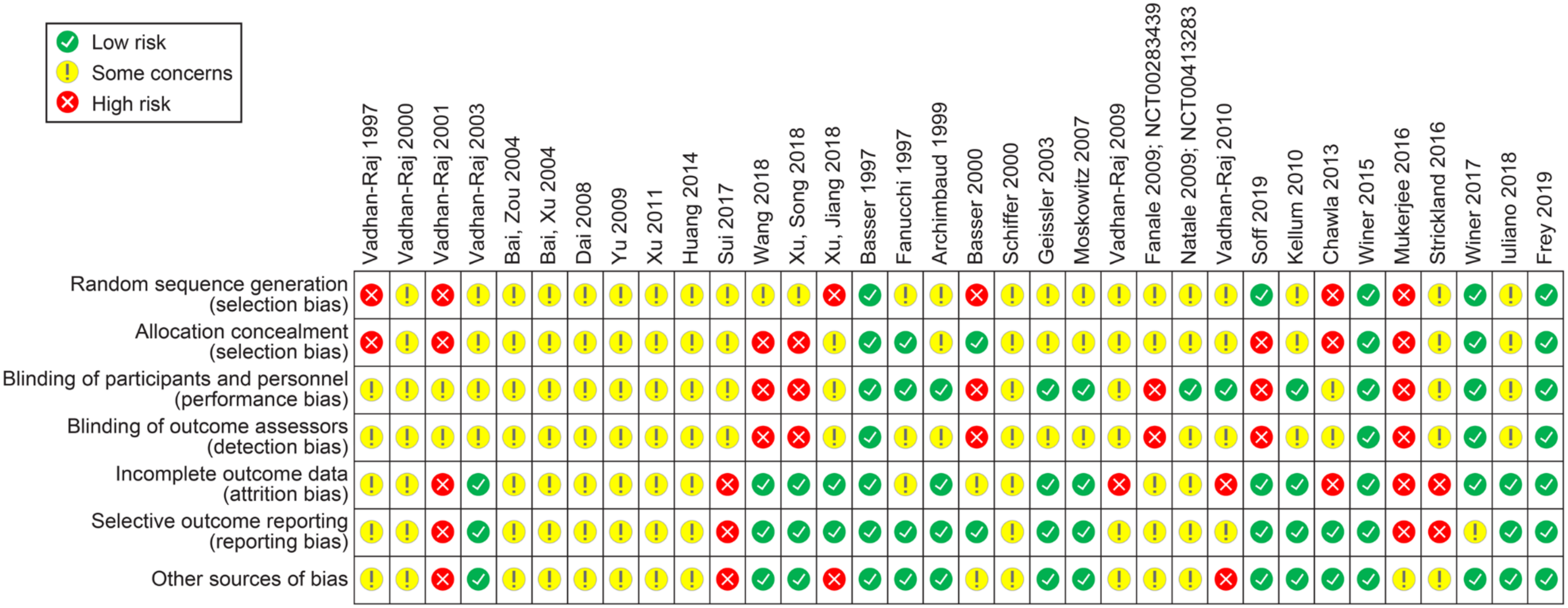

S1 Fig. Risk of bias assessment of clinical trials included in analysis. Clinical trials were assessed using the Cochrane Collaboration's 'Risk of Bias' tool. Studies were evaluated for selection, performance, detection, attrition, and reporting bias and scored as "low risk," "some concerns," or "high risk" of bias for each domain.
